# Intrinsically disordered regions of the Msn2 transcription factor encode multiple functions using interwoven sequence grammars

**DOI:** 10.1093/nar/gkad1191

**Published:** 2023-12-18

**Authors:** Vladimir Mindel, Sagie Brodsky, Aileen Cohen, Wajd Manadre, Felix Jonas, Miri Carmi, Naama Barkai

**Affiliations:** Department of Molecular Genetics, Weizmann Institute of Science, Rehovot 76100, Israel; Department of Molecular Genetics, Weizmann Institute of Science, Rehovot 76100, Israel; Department of Molecular Genetics, Weizmann Institute of Science, Rehovot 76100, Israel; Department of Molecular Genetics, Weizmann Institute of Science, Rehovot 76100, Israel; Department of Molecular Genetics, Weizmann Institute of Science, Rehovot 76100, Israel; Department of Molecular Genetics, Weizmann Institute of Science, Rehovot 76100, Israel; Department of Molecular Genetics, Weizmann Institute of Science, Rehovot 76100, Israel

## Abstract

Intrinsically disordered regions (IDRs) are abundant in eukaryotic proteins, but their sequence-function relationship remains poorly understood. IDRs of transcription factors (TFs) can direct promoter selection and recruit coactivators, as shown for the budding yeast TF Msn2. To examine how IDRs encode both these functions, we compared genomic binding specificity, coactivator recruitment, and gene induction amongst a large set of designed Msn2-IDR mutants. We find that both functions depend on multiple regions across the > 600AA IDR. Yet, transcription activity was readily disrupted by mutations that showed no effect on the Msn2 binding specificity. Our data attribute this differential sensitivity to the integration of a relaxed, composition-based code directing binding specificity with a more stringent, motif-based code controlling the recruitment of coactivators and transcription activity. Therefore, Msn2 utilizes interwoven sequence grammars for encoding multiple functions, suggesting a new IDR design paradigm of potentially general use.

## Graphical Abstract

**Graphical Abstract:**
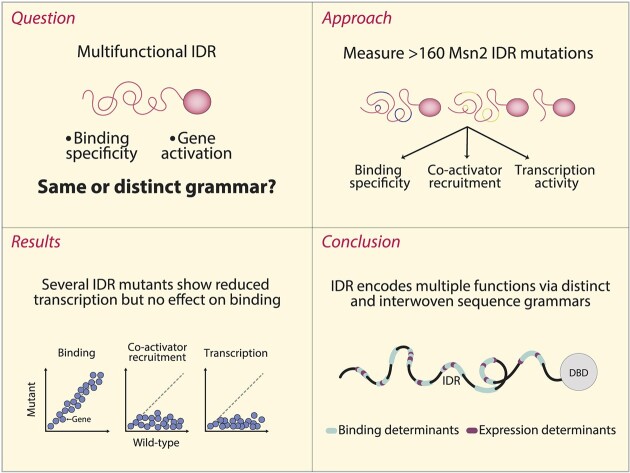

## Introduction

Cells regulate gene expression using transcription factors (TFs) that bind at regulatory regions of specific genes. TFs can be identified by sequence analysis based on their DNA binding domains (DBDs) that belong to known structural families. However, those domains constitute only a small fraction of the TF sequence, while the remaining parts often include long disordered regions (1–6). A limited understanding of the sequence–function relationship of intrinsically disordered regions (IDRs) has left most TF sequences poorly characterized.

IDRs contribute to TF activity in at least two ways. First, activation domains (ADs) that recruit coactivators to induce gene expression reside inside such regions (7–14). Second, IDRs can complement the DBD in directing TF binding preferences along genomes (5,6,15,16).

Intrinsically disordered sequences are often of low complexity, raising the question of how they encode multiple functions. Further emphasizing this question are IDR properties, as revealed by the recent studies. First, determinants of bio-molecular interactions within IDRs are often repeated and redundant, spanning a significant fraction of the sequence (17–22). Furthermore, different functions were attributed to common IDR design features. For example, studying Msn2, we found that the spreading of hydrophobic residues within an otherwise disordered sequence is the key IDR-design feature underlying its role in directing binding preferences (16). Others have reported similar features defining transcription activation domains (10–14), and additional work associated these same patterns with phase separation and formation of protein condensates (23–25).

The similarity of IDR sequence features associated with different functions may point to a common molecular process (16). In the case of Msn2, this suggested to us that the roles of the IDR in dictating binding preferences and in activating transcription are the outcomes of the same molecular interaction. This would be the case, for example, if AD-recruited co-activators also stabilize the binding of the TF at its target promoters.

Here, we examine this hypothesis by directly comparing co-factor recruitment, gene induction capacity, and binding specificity of 161 designed IDR mutants (15,16). We find that determinants of both binding preferences and transcription activity are spread across the long Msn2 IDR. Those determinants, however, differ in two key aspects. First, deletions of short individual segments of the IDR have little effect on binding specificity but readily disrupt transcription activity. Second, binding preferences depend primarily on sequence composition, while transcription activity also requires short sequence motif(s) embedded within the IDR. We conclude that the IDR of Msn2 encodes multiple functions using interwoven grammars of hierarchical complexity, a design that may be of more general use by IDRs involved in intricate functions. We discuss the properties of this design as compared to separated domains used in multi-functional structured proteins.

## Materials and methods

### Budding yeast growth, maintenance and genetic manipulation

All genetic manipulations were performed in the *Saccharomyces cerevisiae* BY4741 background (26) with the MATa his3Δ1 leu2Δ0 met15Δ0 ura3Δ0 genotype using CRISPR. Transformations were performed using the LiAc/SS DNA/PEG method (27). Following validation, the bRA89 plasmid, carrying the CRISPR-Cas9 system, was lost by growth in YPD (yeast extract peptone dextrose) and selection for colonies without bRA89-encoded Hygromycin resistance. Ligation of the gene-specific guide-RNA into the bRA89 plasmid was performed as previously described (17). All strains generated for this study were verified using PCR and gel electrophoresis followed by Sanger DNA sequencing. A detailed description of the strains used in the study is available in Supplementary Tables S1 and S2.

### Cell growth before experiments

Yeast strains were freshly thawed from frozen stock, plated on YPD plates, and grown. Single colonies were picked and grown at 30 C in liquid SD medium (synthetic complete with dextrose) medium overnight, reaching stationary phase, then diluted again into fresh SD medium for the experiment.

### ChEC-seq experiments

The experiments were performed as described previously (28), with some modifications. After the preliminary cell growth described above, the cultures were diluted ∼2 × 10^3^-fold into 5 ml fresh SD media and grown overnight to reach an OD_600_ of 4 the following morning. Cultures were pelleted at 1500*g* for 2 min and resuspended in 0.5 ml buffer A (15 mM Tris pH 7.5, 80 mM KCl, 0.1 mM EGTA, 0.2 mM spermine, 0.5 mM spermidine, 1 × cOmplete EDTA-free protease inhibitors (Roche, one tablet per 50 ml buffer), 1 mM PMSF) and then transferred to 2 ml 96-well plates (Thermo Scientific). Cells were washed twice in 1 ml Buffer A. Next, the cells were resuspended in 150 μl Buffer A containing 0.1% digitonin, transferred to an Eppendorf 96-well plate (Eppendorf 951020401), and incubated at 30°C for 5 min for permeabilization. Next, we added CaCl_2_ to a final concentration of 2 mM to activate the MNase and incubated it for exactly 30 s. The MNase treatment was stopped by adding an equal volume of stop buffer (400 mM NaCl, 20 mM EDTA, 4 mM EGTA, and 1% SDS) to the cell suspension. After this, the cells were treated with Proteinase K (0.5 mg/ml) at 55°C for 30 min. An equal volume of Phenol-Chloroform pH = 8 (Sigma-Aldrich) was added, vigorously vortexed, and centrifuged at 17 000g for 10 min to extract DNA. After phenol–chloroform extraction of nucleic acids, the DNA was precipitated with 2.5 volumes of cold 96% EtOH, 45 mg Glycoblue, and 20 mM sodium acetate at –80°C for > 1 hr. DNA was centrifuged (17 000g, 4°C for 10 min), the supernatant removed, and the DNA pellet washed with 70% EtOH. DNA pellets were dried and resuspended in 30 μl RNase A solution (0.33 mg/ml RNase A in Tris-EDTA [TE] buffer [10 mM Tris and 1 mM EDTA]) and treated at 37°C for 20 min. To enrich small DNA fragments and remove large DNA fragments that might result from spontaneous DNA breaks, DNA cleanup was performed using SPRI beads (Ampure XP, Beckman Coulter). First, a reverse SPRI cleanup was performed by adding 0.8 × (24 μl) SPRI beads followed by 5 min incubation at RT Supernatant was collected, and the remaining small DNA fragments were purified by adding an additional 1× (30 μl) SPRI beads and 5.4× (162 μl) isopropanol and incubating 5 min at RT. Beads were washed twice with 85% EtOH, and small fragments were eluted in 30 μl of 0.1× TE buffer.

### Chec-Seq next-generation sequencing library preparation

Library preparation was performed as described in (29), with slight modifications. DNA fragments following RNase treatment and reverse SPRI cleanup served as an input to end-repair and A-tailing (ERA) reaction, for each sample 20 μl ERA reaction (1 × T4 DNA ligase buffer [NEB], 0.5 mM dNTPs, 0.25 mM ATP, 2.75% PEG 4000, 6U T4 PNK [NEB], 0.5U T4 DNA Polymerase [Thermo Scientific] and 0.5U Taq DNA polymerase [Bioline]) was prepared and incubated for 20 min at 12°C, 15 min at 37°C and 45 min at 58°C in a thermocycler. After the ERA reaction, reverse SPRI cleanup was performed by adding 0.5× (10 μl) SPRI beads (Ampure XP, Beckman Coulter). The supernatant was collected, and the remaining small DNA fragments were purified with additional 1.3 × (26 μl) SPRI beads and 5.4 × (108 μl) isopropanol. After washing with 85% EtOH, small fragments were eluted in 17 μl of 0.1 × TE buffer; 16.4 μl elution was taken into 40 μl ligation reaction (1 × Quick ligase buffer [NEB], 4000U Quick ligase [NEB], and 6.4 nM Y-shaped barcode adaptors with T-overhang (30) and incubated for 15 min at 20°C in a thermocycler. After incubation, the ligation reaction was cleaned by performing a double SPRI cleanup: first, a regular 1.2 × (48 μl) SPRI cleanup was performed and eluted in 30 μl 0.1 × TE buffer. Then instead of separating the beads, an additional SPRI cleanup was performed by adding 1.3 × (39 μl) HXN buffer (2.5 M NaCl, 20% PEG 8000) and final elution in 24 μl 0.1 × TE buffer; 23 μl elution were taken into 50 μl enrichment PCR reaction (1 × Kappa HIFI [Roche], 0.32 μM barcoded Fwd primer and 0.32 μM barcoded Rev primer (30) and incubated for 45 s in 98°C, 16 cycles of 15 s in 98°C and 15 s in 60°C, and a final elongation step of 1 min at 72°C in a thermocycler. The final libraries were cleaned by a regular 1.1 × (55 μl) SPRI cleanup and eluted in 15 μl 0.1 × TE buffer. Library concentration and size distribution were quantified by Qubit (Thermo Scientific) and TapeStation (Agilent), respectively. For multiplexed next-generation sequencing (NGS), all barcoded libraries were pooled in equal amounts, and the final pool was diluted to 2 nM and sequenced on NovaSeq 6000 (Illumina). Sequence parameters were Read1: 61 nucleotides (nt), Index1: 8 nt, Index2: 8 nt, Read2: 61 nt.

### Chec-seq NGS data processing

Raw reads from ChEC-seq libraries were demultiplexed using bcl2fastq (Illumina), and adaptor dimers and short reads were filtered out using cutadapt with parameters: ‘—O 10 –pair-filter = any –max-n 0.8 –action = mask’. Filtered reads were subsequently aligned to the *S. cerevisiae* genome R64-1-1 using Bowtie 2 (31)) with the options’‐‐end-to-end ‐‐trim-to 40 ‐‐very-sensitive’. The genome coverage of fully aligned read pairs was calculated with GenomeCoverage from BEDTools (32) using the parameters ‘-d –5 –fs 1′, resulting in the fragment ends’ position corresponding to the actual MNase cutting sites. For promoter analysis, promoters were defined only for genes with an annotated transcript, as described before (15). The length of each promoter was defined as 700 bp upstream to the transcription start site (TSS) or to the position where a promoter meets another transcript. The signal across each promoter was summed and normalized to the maximal promoter length (700 bp) to calculate the overall promoter binding for each sample.

### Msn2 target definition

To define the Msn2 target genes, first, we standardized the sum of signal on each promoter collected using ChEC-Seq and defined bound promoters as those with Z-score ≥ 3.5. To detect the transcriptionally regulated targets out of the bound ones, data from https://www.ncbi.nlm.nih.gov/geo/query/acc.cgi?acc=GSE234431 was used. This dataset contains whole-genome RNA-seq data of Msn2-Mnase-YFP fusion expressed under a wide range of promoters, validated by flow cytometry. The strains were ordered by their fluorescence measure, and the Spearman rank correlation coefficient was calculated between the fluorescence and respective normalized mRNA levels for every annotated gene, obtaining the correlation coefficient and its respective p-value. A Benjamin-Hochberg FDR procedure filtered all p-values, and those passing the FDR filter were defined as significantly activated by Msn2.

### Med15 target definition

To define the target genes of Med15, we performed a procedure similar to the one above, selecting promoters with *Z*-score ≥3.5. This set was divided into two groups – those common to Msn2 (those overlapping the bound set of Msn2 targets) and those bound only by Med15. Genes not defined as Msn2 targets but showing pre-standardization signals higher than received for Med15 were removed from the analysis.

### Med15 Msn2/4 dependent target definitions

To determine the promoters for which Med15 is dependent on Msn2 or its paralog Msn4, we examined the binding data of Med15 in cells lacking Msn2/4. Genes were defined as Msn2/4 dependent if they were transcriptionally regulated by Msn2 (see Msn2 targets definition above) and had a sum signal of binding lower than 5000 (AU, normalized reads) in the measured binding data of the tested mutant.

### Absolute binding signal

To calculate the absolute binding of the analyzed mutants, we used previously obtained data and compared the cumulative binding signal across all *in-vitro* Msn2 motifs (AGGGG) found in promoters to the relative binding signal at spike-in promoters in the corresponding mutant with spike-in as described previously (16) for a selected subset of Msn2 variants. We then calculated a linear fit between the two parameters and interpolated the spike-in signal and the Msn2 motif binding for Msn2 mutants lacking experimental binding-strength calibration data (Supplementary Figure S2). Finally, the median of the signal on the activated target promoters (defined above) was calculated for each strain and divided by the value of inferred spike-in calibration to obtain an absolute binding value. The data was then normalized between Msn2 WT and Msn2DBD (Msn2 Δ573) as maximal and minimal values proxies. The values received for repeats of the same strain were averaged, and the standard error of the mean overall repeats of a given strain was calculated to visualize the errors.

### Processing and analysis of RNA-seq data

We mapped 50-bp reads of the RNA-seq of every sample to the S. cerevisiae genome (R64 in SGD) using bowtie2 (parameters: -p8 –local –very-sensitive –trim-to 30). After alignment to the genome, samples with <200 000 reads were discarded from the analysis (only samples included in the study with lower than 2e5 reads are of Msn2 ΔMed15 as they showed high inter-repeats correlation) to prevent an artificial enrichment for highly expressed genes. For every sequence, we normalized for PCR bias using the unique molecular identifier (UMI), scoring each position on the genome by the unique number of UMIs it had out of all possible UMIs. For each gene, we summed all the reads aligned to 400 bp upstream, its 3′ end to 200 bp downstream to get that gene's total expression. The number of reads for each sample is normalized to 1e6.

### RNA sample collection, extraction, and sequencing

Six dilutions of cells were grown overnight in liquid SD media in 96 Deep Well plates and then diluted using Tecan Robot. ODs were measured, and 900uL of each culture was collected, choosing the dilution with OD_600_ closest to 0.4 for each strain, and transferred into a new 96-plate. Then, 102uL of H_2_O_2_ in a concentration of 0.00327M was simultaneously added to all wells using a Tecan Robot to a final concentration of 0.3 mM. The plate was incubated at 37°C upon constant shaking for 20 minutes after stress addition to allow transcription of stress-responsive genes. The plate was centrifuged for 60 s at 4000g. The supernatant was removed, and pellets were frozen in liquid nitrogen and stored at −80°C until RNA preparation. mRNA was extracted using a modified protocol of the nucleospin 96 RNA kit (Macherey-Nagel, Duren, Germany). Specifically, cell lysis was done in a 96 deep-well plate by adding 450 μl of lysis buffer containing 1 M sorbitol (Sigma-Aldrich), 100 mM EDTA, and 0.45 μl lyticase (10 IU/μl). The plate was incubated at 30°C for 30 min in order to break the cell wall and then centrifuged for 10 min at 2500 rpm, and the supernatant was removed. From this point, extraction proceeded as in the protocol of nucleospin 96 RNA kit, only substituting β-mercaptoethanol with DTT 1M. RNA libraries were created as described before (33): poly(A) RNA was selected by reverse transcription with a barcoded poly(T) primer. The barcoded DNA–RNA hybrids were pooled and fragmented by a hyperactive variant of the Tn5 transposase. Tn5 was stripped off the DNA by treatment with SDS 0.2%, followed by SPRI beads clean up, and the cDNA was amplified and sequenced with the Illumina NovaSeq 6000 (same parameters as above).

### Med15-Mnase recruitment score

The metric defining Med15 recruitment to the Msn2/4 dependent promoters (defined above) was calculated as Pearson's r correlation coefficient between the respective binding of Msn2 mutant and the strain bearing Med15-Mnase with the same Msn2 mutant. The values received for repeats of the same strain were averaged, and the standard error of the mean over all repeats of a given strain was calculated to visualize the errors. Note that Msn4, the paralog of Msn2, was deleted from all Med15-MNase strains Supplementary Figure S4.

### Expression effect score

The expression data (normalized reads) were normalized to environmental stress response-reduced genes (ESR-reduced) (34) to control for changes in conditions between experiments. First, the median of the ESR-reduced genes was calculated for each sample (M_(x)_), and the overall median value between those samples was then calculated (M_(all)_). The expression vector of activated Msn2 targets (defined above) of each of the analyzed repeats was then multiplied by the fraction of its median stress response to the overall median stress response (M_(x)_/M_(all)_). Next, the median target gene expression difference from ΔMsn2 was calculated for each sample, followed by normalization to the wild-type Msn2 as the maximal value proxy. The values received for repeats of the same strain were averaged, and the standard error of the mean over all repeats of a given strain was calculated to visualize the errors.

### Activation per binding score

To examine the activation efficiency of the examined Msn2 mutants, we investigated the relationship between target gene induction by Msn2 strains and their respective absolute target binding. We defined an activation per binding score as the slope of the line connecting the origin of the graph and the dot on the normalized absolute binding (x-axis) and normalized target expression for each strain (y-axis) graph (Supplementary Figure S3). The calculations were done as follows:


\begin{equation*}Activation\ per\ binding\ score = {\tan }^{ - 1}\left( {\frac{{0 - {E}_i}}{{0 - {B}_i}}} \right)/45\end{equation*}

where *E_i_* is target gene induction, and *B_i_* is the normalized absolute binding of the respective strain. To calculate the error, we used the error propagation function, rendering the following equation:


\begin{eqnarray*} && \Delta Activation\ per\ binding\ score = \left| {\frac{{{E}_i}}{{45B_i^2\left( {B_i^2 + E_i^2} \right)}}} \right|\Delta B_i^{}\nonumber\\ && \quad +\, \left| {\frac{1}{{45{B}_i\left( {B_i^2 + E_i^2} \right)}}} \right|\Delta E_i^{}\end{eqnarray*}

where $\Delta {E}_i$ and $\Delta {B}_i$ are the standard error of the mean of normalized median gene induction and normalized absolute binding, respectively, calculated as above. The activation per binding score of strains showing low absolute target binding and expression effect scores (<0.26) were set to 0 as those are prone to have a high error derived from the parameter calculation. Note that there are no Msn2 mutations in the analysis with low absolute target binding scores and high expression effect scores Supplementary Figures S3, S5, S7.

### Activation prediction using PADDLE

**Figure 1.**
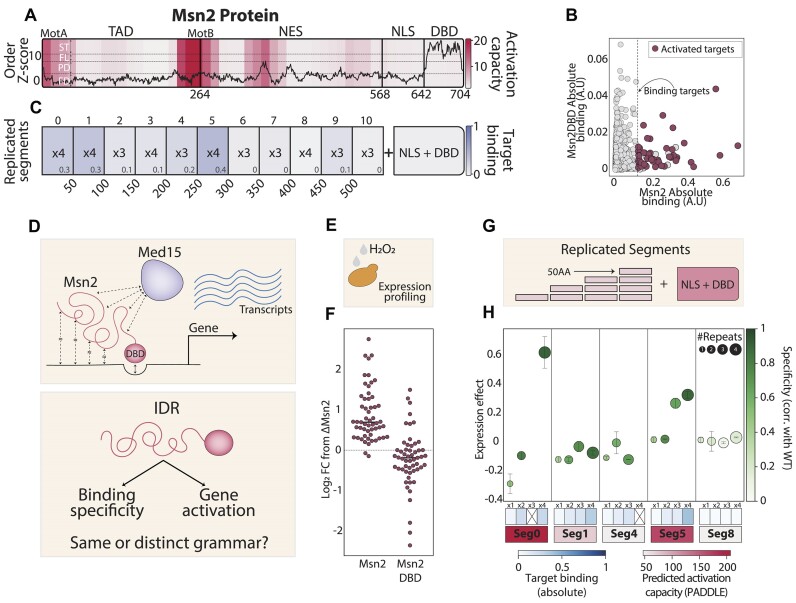
The Msn2 IDR contains multiple predicted activation domains (ADs): (**A**) ADs within the Msn2 protein sequence: The disorder tendency at each position of the Msn2 AA sequence is shown by the black solid line, as predicted by ADOPT (43). The dashed lines represent the different ADOPT-defined categories (FD = fully disordered, PD = partially disordered, FL = flexible and ST = structured). The transcription activation capacity of each 53AA region, as measured in (14), is shown in color-code (methods). The different Msn2 domains, including two motifs (MotA and MotB) previously found to exhibit expression capacity, are also annotated (37). (**B**) Msn2 binding locations along the genome depend on regions outside its DBD: the binding profiles of Msn2 and a mutant lacking its non-DBD (DBD-only) were measured using ChEC-seq (28). The respective promoter binding signals are shown calibrated using an external control (methods), where each dot represents a promoter. The dashed line separates Msn2-bound promoters, and the subset of Msn2-induced targets is colored (methods). (**C**) Msn2 contains multiple binding determinants outside its DBD: each indicated segment was fused to the Msn2 DBD in the specified number of tandem repeats. Next, genomic binding profiles were measured, as described (Supplementary Figure S2, methods). Shown in color code and numeric representation (bottom-right corner of each square) is the absolute target promoter binding for the respective fusion (methods). The data was obtained from (16). (**D–F**) Quantifying gene induction capacity of Msn2 mutants: Msn2 induces its target genes by recruiting the Med15 coactivator. Both the promoter selection and the subsequent gene induction depend on the highly disordered Msn2 non-DBD (D, top). We wish to define the sequence grammar directing binding specificity and transcription activity (D, bottom). As we previously mapped the binding preferences of >160 Msn2 mutants, we here complement this data by measuring their transcription activity, which we quantified based on target gene induction following stress exposure (E, H_2_O_2_, methods). The robust induction seen in cells carrying the full Msn2 was abolished in cells carrying the DBD-only mutant, as demonstrated in (F), where each dot represents a target gene, and the y-axis shows the normalized log_2_ fold-change difference from a strain deleted of Msn2 (see methods for normalization). (**G, H**) Predicted ADs can induce Msn2 target genes when fused in tandem repeats to the Msn2 DBD: we selected five 50AA Msn2 segments of varying PADDLE (14) predicted AD activity and fused them in different repeat numbers to the Msn2 DBD (G). Shown in (H) is the normalized median induction of the Msn2-target genes following stress exposure (methods). The similarity in binding specificity, measured by the respective correlation of promoter selection to the wild-type Msn2, is shown in color code (green, methods). Error bars represent the standard error of the mean between repeats (SEM, methods). The target binding of each fusion (blue, as in C) and the PADDLE-predicted activation (red, calculated for a single segment) is shown as a color code on the bottom. Segments are numbered as in (C).

To calculate the predicted activation capacity of the Msn2 mutants, a machine-learning algorithm termed PADDLE was used (14). The vector containing the predicted activity of all calculated 53AA segments was summed to obtain the predicted activity of each mutant or plotted as it is in Supplementary Figures S5 and S11. For Figure 1A, the experimental data, not the prediction, was used to show the distribution of activation determinants along the Msn2 sequence. The data were smoothed by the mean of the centered rolling window of three data points and plotted as a heatmap.

## Results

### Multiple regions within the Msn2 IDR can activate gene expression when present in tandem repeats

Msn2 is a zinc-finger TF that induces stress genes in budding yeast (35,36). Its non-DBD sequence is 642 amino acids (AAs) long, of which 99.8% are predicted to remain fully or partially disordered (Figure 1A) (15). Previously, we found that removing this region shifts Msn2 binding away from its target promoters (Figure 1B). Using extensive internal deletions and truncations of the Msn2 non-DBD sequence we mapped this effect to the cumulative action of multiple disordered segments (15). Others explored the role of this IDR in gene activation, including earlier studies that defined two short motifs (MotA and MotB) needed for Msn2 to induce HSP12—one of its target genes (37), and a more recent, systematic analysis that identified multiple 53AA segments within this IDR that can function as ADs (14) (Figure 1A).

When examining these data, we noted an overlap between regions we identified as contributing to target binding and those classified as ADs (Figure 1C), suggesting that determinants of binding specificity and transcription activity co-localize along the Msn2 IDR (Figure 1A, C, D).

The previous assay that systematically classified ADs was based on a DBD and a reporter, both unrelated to Msn2 (14). We wished to verify the capacity of those classified ADs to induce the Msn2 target genes. For this, we considered profiling the genome-wide expression patterns under stress conditions (Figure 1E, methods). Upon stress exposure, Msn2 is translocated into the nucleus, binds to stress gene promoters, and induces their expression. As expected, this stress-induced program is lost in the Msn2 DBD-only mutant lacking the long IDR (Figure 1F).

To measure the Msn2-related AD capacity, we selected five 50AA segments of different PADDLE-predicted activity and fused them to the Msn2 DBD (Figure 1G). We knew from previous studies that single fusions of those short segments to the DBD do not retrieve Msn2 binding at its target promoters and, indeed, none of those rescued stress-gene induction (Figure 1H). Note that here, and in all analyses below, we quantify binding specificity using correlation (Pearson), comparing the sum of signal over all promoters between the measured mutant and the wild type Msn2 (methods). Promoter binding specificity was gradually retrieved when fusing tandem repeats of most segments, and two of those also induced the Msn2 target genes. Notably, segments showing AD activity were given the highest PADDLE-predicted activation scores and overlapped with the MotA and MotB motifs (37) (Figure 1H) (16). Still, this rescue of target induction was partial and only observed when fusing 3–4 repeats of the same segment. We conclude that the Msn2 IDR contains multiple regions classified as ADs, capable of inducing Msn2 target gene expression, but only when present in multiple repeats.

### Msn2 transcription activity depends on multiple IDR determinants

**Figure 2.**
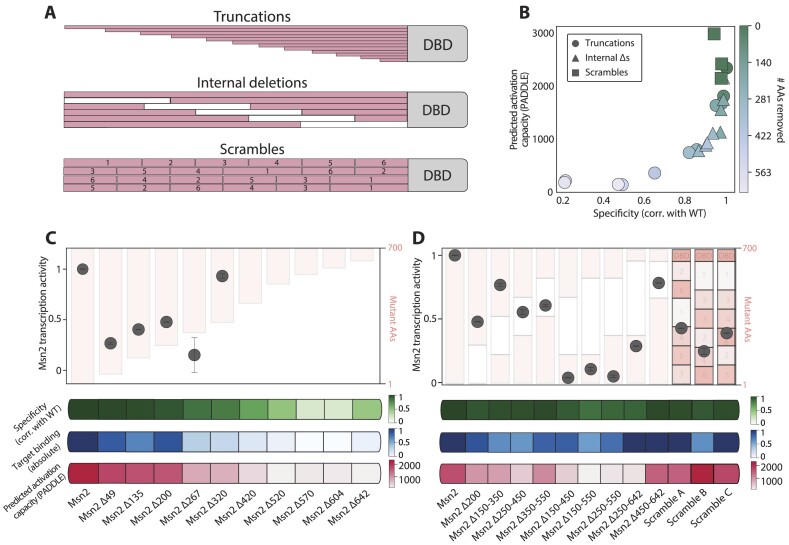
The Msn2 transcription activity is sensitive to multiple deletions throughout the IDR: (**A**) Msn2 IDR deletion and scrambling mutants: we used three sets of mutants to define the IDR regions needed for the Msn2 transcription activity. Those included a series of truncations (top), where we gradually deleted ∼50AAs starting from the Msn2 N-terminus (top), internal deletions of 200 or more AAs spanning across the Msn2 non-DBD (middle), and scrambling of 100AA Msn2 segments (bottom). The binding preferences of all those Msn2 mutants were previously measured (15). We now supplemented this data by measuring their capacity to induce the Msn2 target genes, as in Figure 1E–G. (**B**) Predicted activation capacity of Msn2 gradual truncation mutants correlates with their measured binding at target promoters: we used the PADDLE predictor to define the expected activation potential of each mutant (methods) and show it as a function of the correlation of promoter selection between the measured mutant and the wild-type Msn2, denoted as specificity. Data taken from (15). (**C**) Msn2 transcription activity is lost upon the deletion of an N-terminal AD: shown on top is the transcription activity for each mutant. The transcription activity of strains found in the high error area was not calculated (Supplementary Figure S3, red ‘X’ markers). Error bars represent the SEM between repeats (methods). The background color captures the truncation length. Shown in green for each mutant is the similarity of binding specificity to the wild type, which is defined by the correlation of promoter selection with the full Msn2. Shown in blue is the absolute binding strength at target promoters for each mutant as in (Figure 1C). PADDLE-predicted activity is shown in pink at the bottom. (**D**) Msn2 transcription capacity is sensitive to internal deletions and scrambling, showing little effect on binding specificity: same as (C) for the indicated internal deletions and scrambled Msn2 variants.

Our finding that IDR segments, which can induce reporter genes when fused to an unrelated DBD, are still insufficient for inducing Msn2 target genes motivated us to re-examine the spread and effects of activation determinants within the full Msn2 sequence. For this, we analyzed three series of Msn2 mutants (15): Truncations that shortened the IDR through gradual ∼50AA steps, internal IDR deletions of 200AAs (or more), and scrambling of ∼100AA IDR segments (Figure 2A). We previously mapped the genomic binding of all those mutants, and now noted that their PADDLE-predicted activation capacity corresponds well with our measured binding specificities (Figure 2B).

We next measured experimentally the capacity of each mutant to induce target gene expression, as above. Examining the expression patterns of each target gene across all strains analyzed in this study revealed that the capacity of a given IDR mutant to activate gene transcription is uniform across all targets (Supplementary Figure S1). To quantify transcription activity, we normalized the median fold change of the expression of all target genes by the absolute Msn2 target binding strength obtained through external calibration (Supplementary Figures S2, S3, methods). Therefore, our measure of Msn2 activity, used in all analyses below, is in units of gene induction per Msn2-binding. Note that this measure becomes inaccurate at low binding levels. None of those low-binding mutants, however, displayed considerable gene induction, as expected (Supplementary Figure S4A, B).

Examining these normalized activity scores revealed that the Msn2 transcription activity was dramatically reduced at the first 50 AA truncation, which had no apparent effect on binding specificity (Figure 2C and Supplementary Figure S4A–C). The transcription activity remained low in subsequent truncations. The low target binding of these truncations made it difficult to assess whether additional regions within the Msn2 IDR contribute to its transcription activity and likely explain some non-monotonic effects seen in longer truncations (e.g. Δ267 versus Δ320).

We next tested the internal truncations, all of which retained high specificity. Most of those deletions reduced (Δ200AA) or even abolished (>Δ200AA) transcription activity (Figure 2D and Supplementary Figure S4A–C). A similar reduction was also seen upon scrambling of ∼100AA segments within the Msn2 IDR, which again had little effect on binding specificity and strength (Figure 2D). Of note, the loss of activity of the latter set of mutants contrasted their PADDLE-predictions, which remained high in all three cases. Closer examination of those predictions, however, revealed the breaking of ADs and changes in their distribution across the sequence (Supplementary Figure S5), suggesting that within the long IDR sequence, transcription activity depends not only on the cumulative strengths of ADs but also on their arrangement within the sequence. We conclude that transcription activity is highly sensitive to deletion or even scrambling of multiple individual segments within the Msn2 IDR while having little effect on its promoter binding specificity.

### Recruitment of the Med15 coactivator by Msn2 requires multiple intrinsically disordered regions

**Figure 3.**
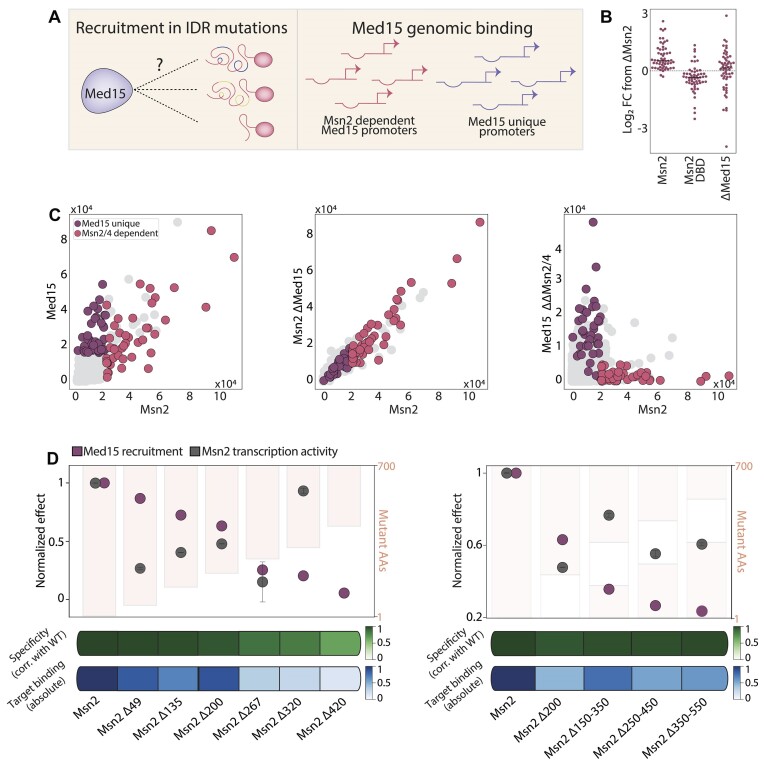
Med15 recruitment to Msn2 target promoters is sensitive to multiple deletions throughout the IDR: (**A-C**)Med15 is recruited to Msn2 target promoters: In wild-type cells, Med15 is recruited by Msn2. We wish to define how the different Msn2 mutations affect this recruitment (A). This recruitment is essential for the transcription activity of Msn2, rendering it an additional reporter for quantifying the effect of Msn2 IDR mutants (B, presentation as in Figure 1F, for the indicated strains). The scatter plots in (C) show the similarity of the binding profiles of Med15 and Msn2 (left), the lack of effect of a Med15 deletion on the binding of Msn2 (center), and the loss of Med15 binding upon the deletion of Msn2 and Msn4 (right). Each point is a promoter positioned according to the binding signal received for the respective strains using ChEC-Seq. (**D**) Med15 recruitment to Msn2 target promoters is sensitive to Msn2 internal deletions: same as Figure 2C above, adding Med15 recruitment as defined by the correlation of target promoter binding between the respective Msn2 variant and Med15 measured in the same strain (Supplementary Figure S6, methods, burgundy).

The loss of the transcription activity of Msn2 upon removal of individual segments surprised us, given that multiple classified ADs were retained within the sequences (14). We tested this further by measuring Med15 recruitment as an additional reporter of the transcription activity of Msn2 (Figure 3A). Med15 is a mediator tail component that serves as a key coactivator of most budding yeast TFs, including Msn2 (14,38–40). When tested in our setup, Med15 deletion greatly reduced the capacity of Msn2 to activate its target genes while having no effect on its binding specificity (15) (Figure 3B, C). Further, in wild-type cells, Med15 localizes to Msn2 target promoters in a manner that requires either Msn2 or its paralog Msn4 (41) (Figure 3C). Med15 binding can therefore serve as an additional measure of Msn2-dependent transcription activity.

As in our previous studies, we mapped Med15 binding along the genome using the spatially resolved ChEC-seq method (28), in which the protein of interest (Med15 in our case) is fused to an MNase, enabling triggered cleavage of proximal DNA through a short (30′) calcium pulse. Collecting, sequencing, and mapping the cleaved fragments to the genome provides a spatially resolved profile of DNA-bound locations (methods). Using this data, we quantified the capacity of each Msn2 variant to recruit Med15 by measuring the binding similarity (correlation) of Med15 and Msn2 in each strain (Supplementary Figure S6, methods). Note that this measure indeed captures the expected reduction in Msn2-dependent Med15 binding peaks observed in the Msn2 DBD-only mutant (Supplementary Figure S7).

Testing the effect of the different Msn2 truncations and internal deletions on the capacity to recruit Med15 revealed a gradual decrease in recruitment with increasing truncations. Med15 recruitment was largely reduced, or even lost, in internal deletions that had little or no effect on the Msn2 binding specificity (Figure 3D). Therefore, both gene induction and Med15 recruitment require cooperative action of multiple determinants that spread across the Msn2 IDR.

### Replacing IDR acidic residues with positively charged ones abolishes transcription activity, contrasting a limited change in binding specificity

**Figure 4.**
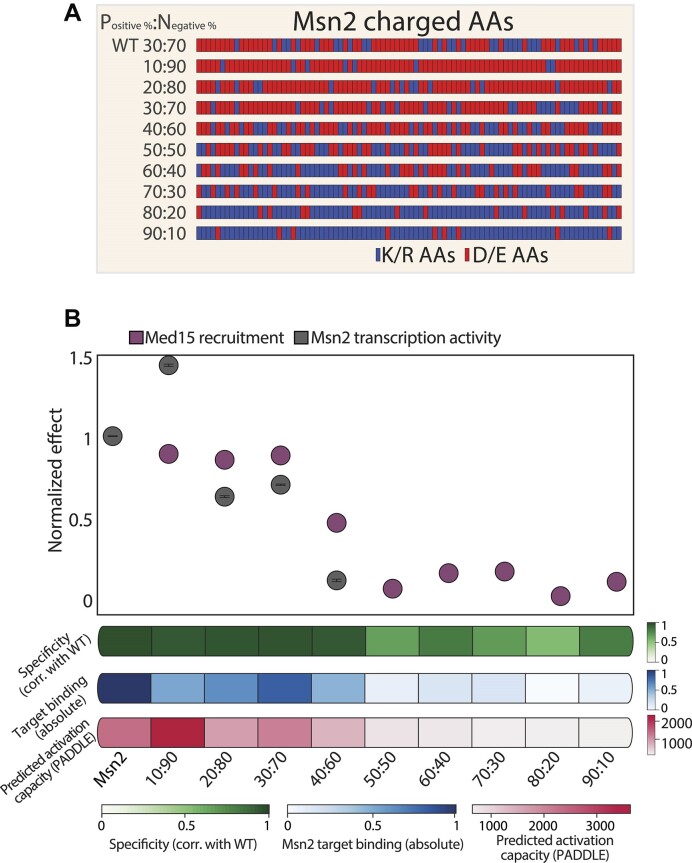
Replacement of acidic IDR residues with basic ones abolishes the Msn2 transcription activity while having little effect on its binding specificity: A series of IDR mutants of gradually varying charge was designed by repositioning different ratios of acidic/basic residues at the original DEKR positions (**A**, showing only the set of 90 charged AA found within the Msn2 IDR). Shown on (**B**, top) are The Med15 recruitment (burgundy) and the transcription activity (gray) of each mutant. The binding specificity (green), target binding (blue), and PADDLE prediction (pink) are shown as color maps at the bottom.

Truncating Msn2 not only removes the regions capable of transcription activation but also drastically shortens the protein. To further probe the contribution of the various regions to the transcription activity while retaining the long IDR, we perturbed the acidic or hydrophobic residues essential for AD activity (14) using three sets of IDR mutants: One in which we changed the overall charge of the IDR while maintaining the number and locations of charged residues, and two in which we removed hydrophobic AAs by segments of increasing lengths starting from either the N or the C-terminal ends of the IDR (Figure 4A, Supplementary Figure S8). In all three series, the effects on gene induction or Med15 recruitment were more severe than on promoter binding specificity (Figure 4B, Supplementary Figure S8, and Supplementary Figure S9).

### Ortholog comparison reveals rapid divergence of transcription activity contrasting the conservation of binding specificity

**Figure 5.**
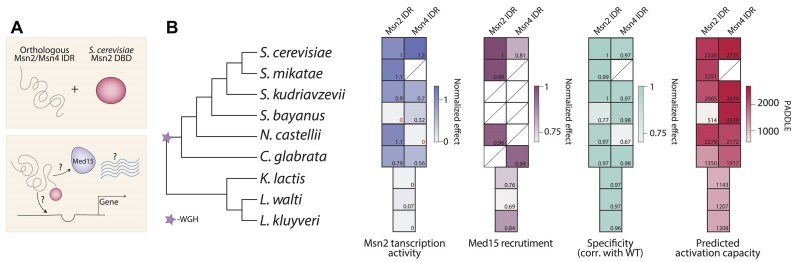
Msn2 transcription activity diverges rapidly amongst orthologs: (**A, B**) We examined the divergence of the Msn2 non-DBD amongst orthologs by replacing the corresponding Msn2 sequence within *S. cerevisiae*, as shown in the scheme, and profiling their binding specificity, transcription activity, and Med15 recruitment (A). Shown in (B, left) is a phylogenetic tree of the species from which the orthologous IDRs were taken. The star indicates a whole genome hybridization (WGH) event that occurred ∼100 million years ago (44). Two IDRs, corresponding to Msn2 and its paralog Msn4, were examined if present. Shown in (B, right) for each ortholog is the transcription activity (blue; strains found within the high-error area indicated by red zeros), Med15 recruitment (burgundy), similarity of promoter binding preference to WT (green), and the PADDLE activity prediction (pink). The values indicated by colors are also found on the bottom right corners of each square. Missing data is indicated by diagonal lines (‘/’). Species tree is according to (45).

Finally, as a complementary viewpoint, we compared transcription activity amongst IDR orthologs from other yeast species. For this, we selected species of different evolutionary distances that retained similar-sized IDRs and mostly conserved binding specificity (15). Testing the transcription activity of those orthologs revealed a considerable divergence, with several orthologs showing a substantial or even full loss of gene induction (Figure 5, Supplementary Figure S10A). Notably, these effects were predicted by the associated PADDLE profiles. In fact, the PADDLE profiles of orthologs that retained transcription activity strongly resembled that of the *S. cerevisiae* Msn2, including the positioning and distances between the activation peaks (Supplementary Figure S11). By contrast, no apparent activation peaks were observed in the PADDLE profiles of orthologs that have lost transcription activity. It is also notable that those orthologs showing loss of transcription activity and PADDLE signal did retain a reduced but considerable capacity to recruit Med15. The basis of this difference and whether or how those orthologs activate gene expression within their natural species remains unclear. We conclude that determinants of binding specificity are highly redundant, while those encoding transcription activation are mutually required, suggesting their cooperation in coactivator recruitment.

### Msn2 transcription activity relies on sequence motifs embedded within its IDR

**Figure 6.**
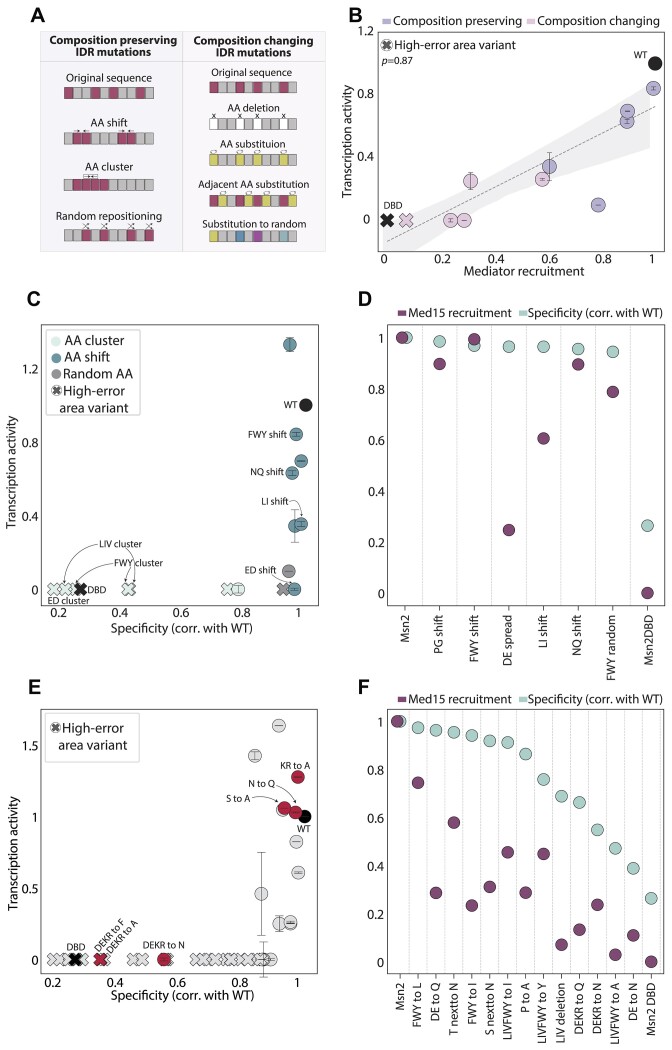
Msn2 transcription activity is sensitive to sequence mutations having little effect on target promoter binding: (**A, B**) Coactivator recruitment correlates with transcriptional activity: To decouple the interwoven effects of the total IDR composition and short sequence motifs, we examined two types of designed Msn2 IDR mutants: Those that preserve the overall sequence composition (A, left) and those that change it (A, right). Shown in (B) is the transcription activity of those mutants as a function of Med15 recruitment for a selected subset of strains for which both data types were collected. Linear regression with a shaded area representing the confidence interval is also shown (α = 0.05, methods, strains marked by an ‘X’ are found within the high-error area, and their transcription activity was set to zero, Supplementary Figure S1). (**C–F**) Msn2 transcription activity is sensitive to IDR mutations not influencing binding specificity: Shown is a comparison of the transcription activity (C, E) or Med15 recruitment (D, F) to the similarity in binding specificity of the different Msn2 variants to the wild-type TF. The mutants are classified as composition preserving (C, D) or composition changing (E, F). Strains marked by an ‘X’ are found within the high-error area; therefore, their transcription activity was set to zero. Marked in red (E) are strains discussed in the main text.

The involvement of IDRs in specific molecular interactions is often attributed to short sequence motifs. To distinguish the role of such motifs, we tested the transcription activity of both composition-changing and composition-preserving IDR mutants (Figure 6A). Note that the latter class retained all original AAs but shifted their positions through clustering together or locally shifting an AA of choice, thereby abolishing potential sequence motifs. We tested the effect of those mutations on Med15 recruitment and/or gene induction using the assays described above. Note that the change in those two transcription-related aspects was highly correlated when applied to the same mutants (Figure 6B).

Focusing first on the composition-preserving mutants, we previously showed that clustering of hydrophobic or charged residues abolishes Msn2 nuclear localization and binding at target promoters (16). Indeed, none of those mutants were capable of stress gene induction (Supplementary Figure S10B). Contrasting the AA-clustering, locally shifting AA locations had little or no effect on the binding specificity of Msn2 (16). Still, several of those mutants failed to induce gene expression or recruit Med15 (Figure 6C, D). Among those, shifting the acidic (ED) or aliphatic (LI) residues had the most significant effects, fully abrogating transcription, while shifting polar residues (NQ) had a somewhat lesser effect but still showed detectable consequences. Notably, shifting the aromatic residues (FWY) was also of intermediate effect, suggesting that they act through general composition-based interactions rather than sequence motifs. We conclude that Msn2 transcription activity is sensitive to the precise positioning of certain residues, while binding specificity is largely robust to such changes.

### Sensitivity of Msn2 transcription activity to IDR composition

We next examined the role of the AA composition of the IDR by considering mutants in which same-type residues were deleted or replaced (Figure 6A, right). A substantial fraction of these mutants lost their binding specificity to the Msn2 target promoters, and those invariably abolished gene induction and Med15 recruitment, as expected (Figure 6E, F, Supplementary Figure S10C). Examples included replacing all charged residues (DEKR→A, F) or all hydrophobic residues (LIVFWY→A).

We were, again, more interested in mutants that retained the wild-type promoter binding specificity. Some of those were transcriptionally active, including N→Q, S→A, and KR→A. Others, however, have lost transcription activity, including the substitution of all charged residues (e.g. DEKR→N/Q, Figure 6E, F) or inter-hydrophobic replacements (e.g. LIVFWY→I, and LIVFWY→Y). Together, these results revealed a high sensitivity of transcription activity to sequence changes having little effect on promoter binding specificity.

## Discussion

The interest in the sequence-function relations within IDRs has increased in recent years as the accumulated data demonstrated the role of disordered protein regions in diverse cellular functions. In this study, we examined the capacity of a single IDR to encode multiple functions. Structured proteins can carry several tasks independently using separate folded domains, but this spatial separation is difficult to achieve within an IDR, where information tends to spread across the entire sequence.

We focused on Msn2 as its long IDR is involved in two well-characterized functions: Binding specificity and transcription activity. Our analysis pointed to two key features distinguishing the respective sequence codes. The first is the extent of redundancy. We previously showed that binding specificity is largely invariant to the deletion of large (∼200AA) regions across the Msn2 IDR. We also expected this to be the case for transcription activity, as the IDR contained multiple different segments that were shown to act as ADs in a reporter assay and those extended beyond the two initially described motifs (37). Contrasting this expectation, we found that multiple ADs embedded within the Msn2 IDR are required for its transcription activity. We demonstrated this both when fusing individual segments to the Msn2 DBD and when deleting internal segments within the Msn2 IDR, which led to the loss of transcription activity. Of note, this rapid loss of function was not due to changes in IDR length or disorder tendency, as it was also detected in mutants in which we maintained the length of the IDR but changed the fraction of acidic residues or when we only moderately changed the IDR length (∼20% of total) through removing hydrophobic residues.

Also showing rapid activity loss were Msn2 non-DBD orthologs, which we tested by swapping the *S. cerevisiae* counterparts. Almost all tested orthologs retained the binding to Msn2 target promoters, but only some have also induced the Msn2 target genes. Given the high conservation of ADs, we did not expect this result, yet the loss of transcription activity was, in fact, well-predicted computationally by PADDLE. The basis of this evolutionary function-divergence remains to be tested.

The sequence codes associated with binding specificity and transcription activity also differed by the role of sequence motifs. We previously found that local AA shifts, designed to abrogate such motifs, are of little consequence on the binding specificity of Msn2. By contrast, such mutations readily disrupted its transcription activity. Most dramatic here were local changes in the locations of aliphatic (LI) or acidic (DE) residues, both of which reduced gene induction while having minimal impact on target binding. Interestingly, a shift in aromatic FWY residues had a lesser effect. This could be the result of their lower abundance (4% FWY of the IDR sequence compared to 12% LI and 8% DE) but could also suggest that aromatic residues contribute to transcription activity more through sequence composition, while Leucine/Isoleucine is required as a part of a sequence motif itself. In this context, we note that the LxxLL motif implicated in many ADs is only present once within the long IDR of Msn2 (14).

**Figure 7.**
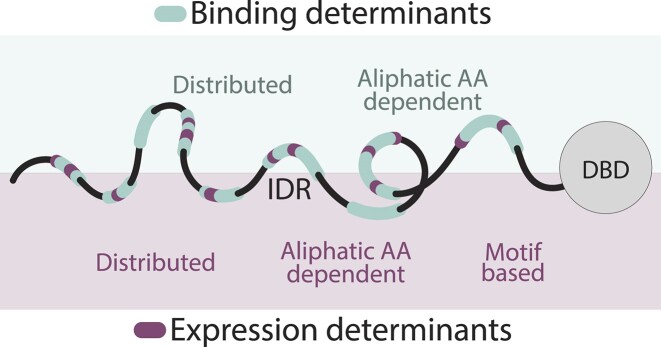
Model: Interwoven IDR sequence grammars: The capacity of the Msn2 IDR to direct genomic binding specificity depends primarily on its sequence composition. In contrast, its ability to induce target gene expression, which again depends on determinants spread across the IDR, requires more stringent sequence motifs.

Our data suggest a model in which the Msn2 IDR can encode multiple functions using interwoven and hierarchical sequence grammars (Figure 7). Such a design is based on three key features. First, rather than being spatially separated, as in structured proteins, the information encoding for the two functions is spread across long and overlapping regions. Second, an overall sequence composition in which hydrophobic residues are exposed within an otherwise hydrophilic environment appears to be commonly required. Finally, this general design is refined, to varying extents, through sequence motifs that favor specific interactions with partners of interest, such as Med15 and/or other coactivators. It is notable that a similar design, combining sequence composition and short motifs, was defined recently in accounting for the essentiality of the general TF Abf1 (42). Further studies will test the generalization of this model to IDRs other than that of Msn2.

In closing, one of our motivations in initiating this study was to test whether the Msn2 IDR stabilizes binding at its target promoters through interactions with the general transcription machinery. The similarity in the inferred molecular codes has made this hypothesis plausible. Our current findings rule out this possibility, at least with respect to the immediate candidate (Med15). We are, therefore, still left with the question of what explains the role of IDRs in binding specificity. Interactions with other TFs that co-localize to the same promoters are an option, although our recent studies testing this also question this interpretation (41). Direct interactions with nucleosomes or DNA are other possibilities to be tested in future studies.

## Supplementary Material

gkad1191_Supplemental_Files

## Data Availability

The code describing the analysis presented in the manuscript is available at https://doi.org/10.5281/zenodo.10043642. The sequencing data is available at https://www.ncbi.nlm.nih.gov/geo/query/acc.cgi?acc=GSE239884.
