# Supplementary material for: Intrinsically disordered regions of the Msn2 transcription factor encode multiple functions using interwoven sequence grammars
Source: Nucleic Acids Res. 2023 Dec 18;52(5):2260–72. doi: 10.1093/nar/gkad1191 (PMC10954448; doi:10.1093/nar/gkad1191)
Supplement: gkad1191_Supplemental_Files [file gkad1191_supplemental_files.zip › SupplementalMaterials.pdf]

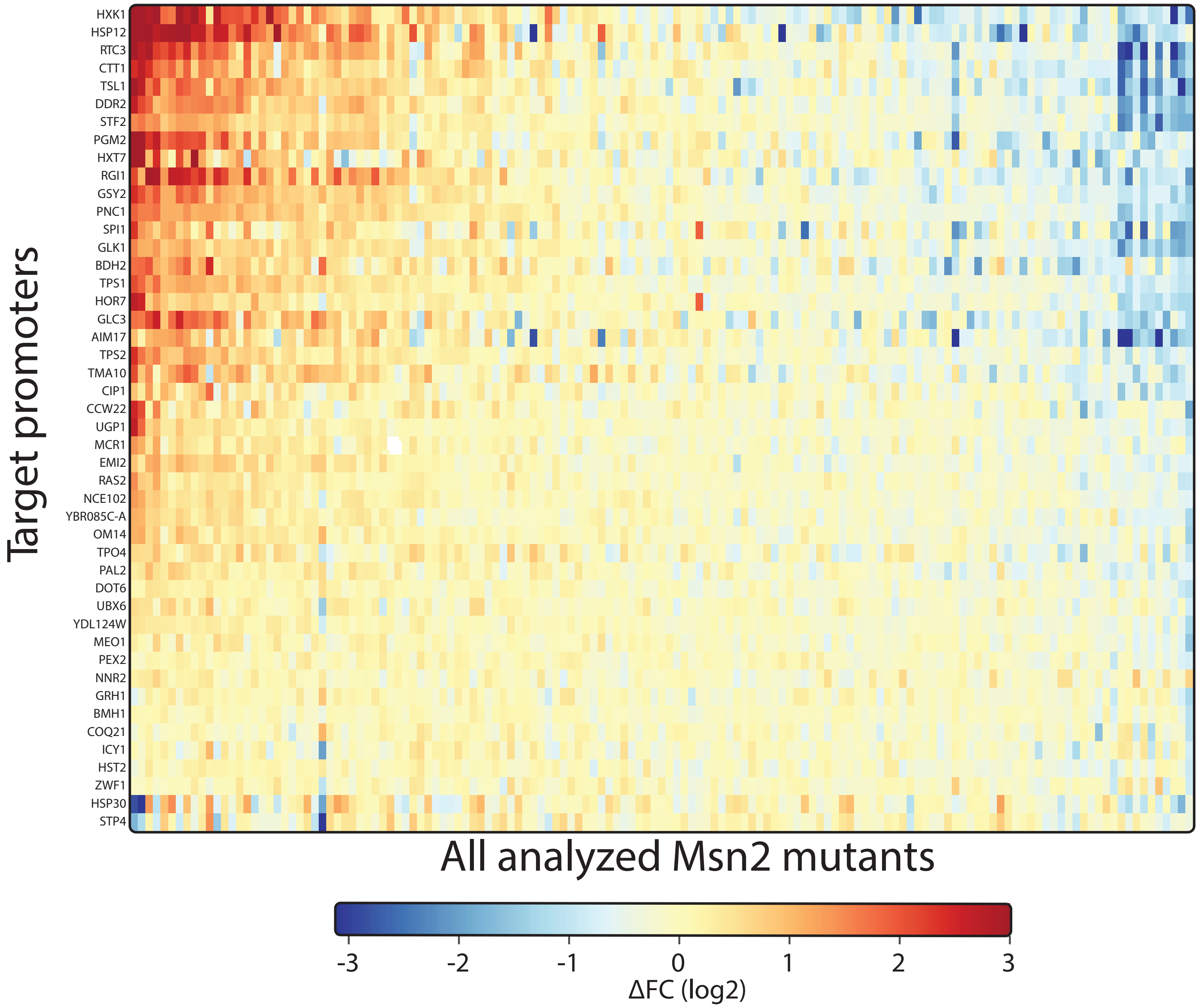
Target promoters
HXK1
HSP12
RTC3
CTT1
TSL1
DDR2
STF2
PGM2
HXT7
RGI1
GSY2
PNC1
SPI1
GLK1
BDH2
TPS1
HOR7
GLC3
AIM17
TPS2
TMA10
CIP1
CCW22
UGP1
MCR1
EMI2
RAS2
NCE102
YBR085C-A
OM14
TPO4
PAL2
DOT6
UBX6
YDL124W
MEO1
PEX2
NNR2
GRH1
BMH1
COQ21
ICY1
HST2
ZWF1
HSP30
STP4
All analyzed Msn2 mutants
-3
-2
-1
0
1
2
3
ΔFC (log2)

Figure S1. *Dynamics of the Msn2 target genes across all analyzed strains*

Shown is a heatmap representing the expression dynamics of the Msn2 target genes across all analyzed strains. Noisy target genes were removed from this analysis (<4 log2 of 10^6 normalized counts, methods), leaving 46/55 genes out of the original dataset. Shown is the log2 fold change from the median of all analyzed strains (161). Strains are positioned according to the median effect across the analyzed set of genes, and the genes are sorted by the difference between the medians of the 5 highest-effect and 5 lowest-effect strains. Note that the capacity of a given IDR mutant to activate transcription is uniform across all target genes.

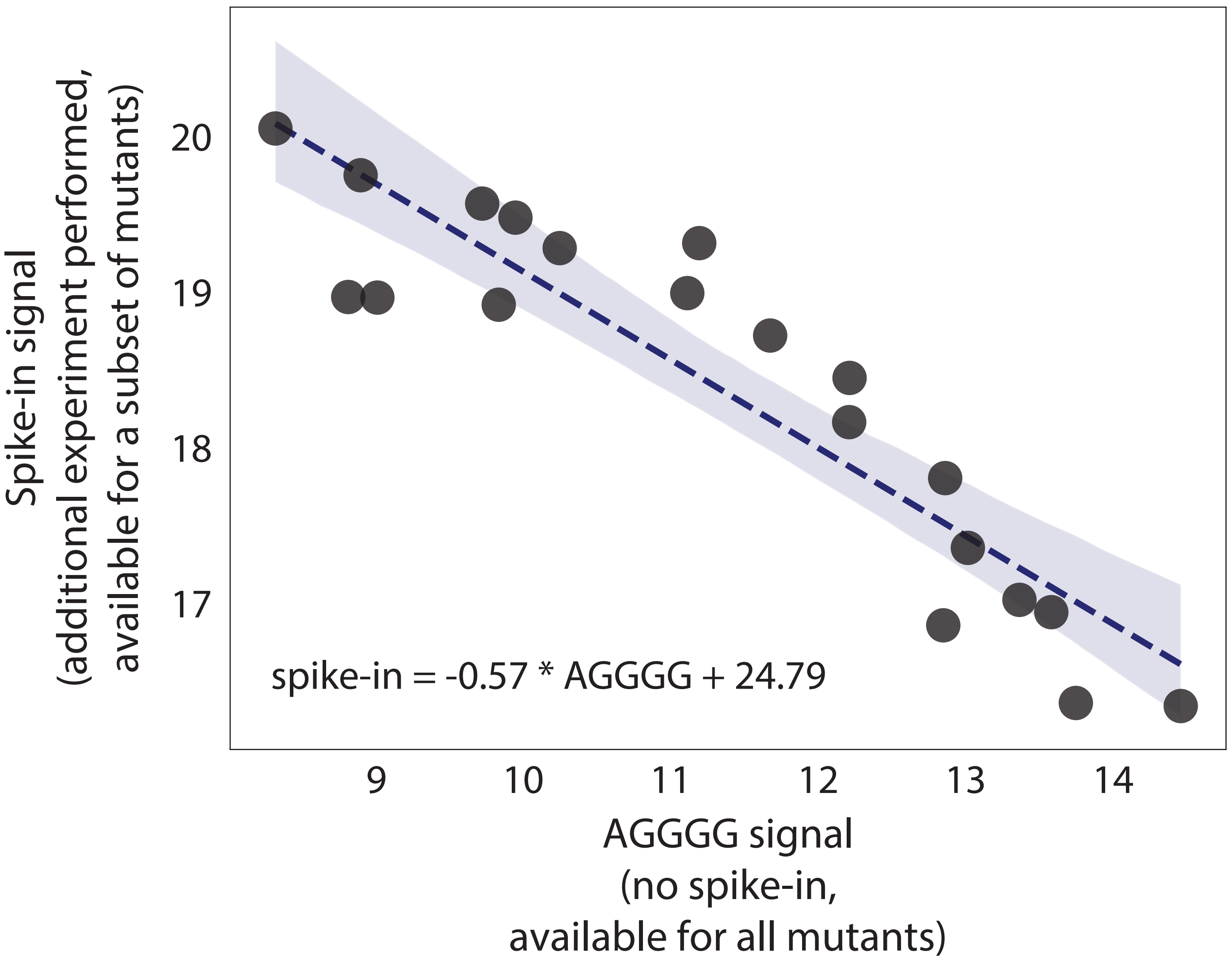
Spike-in signal
(additional experiment performed,
available for a subset of mutants)
20
19
18
17
spike-in = -0.57 * AGGGG + 24.79
9
10
11
12
13
14
AGGGG signal
(no spike-in,
available for all mutants)

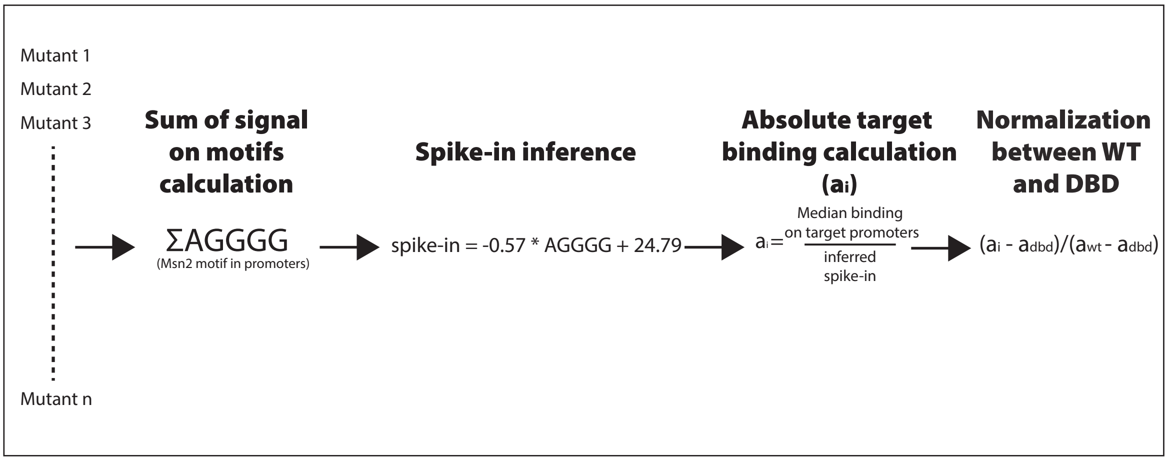
Mutant 1
Mutant 2
Mutant 3
Mutant n
Sum of signal on motifs calculation
ΣAGGGG
(Msn2 motif in promoters)
Spike-in inference
spike-in = -0.57 * AGGGG + 24.79
Absolute target binding calculation (ai)
ai =
Median binding on target promoters
inferred spike-in
Normalization between WT and DBD
(ai - adbd)/(awt - adbd)

Figure S2. *Absolute binding to target promoters*

Two datasets were used to obtain an absolute binding measure for all Msn2 mutants. The first dataset contains regular ChEC-seq data (methods) for a subset of strains analyzed in this study. From this set, we calculated the sum of signal on Msn2 preferred *in-vitro* motifs that reside in promoters for each Msn2 variant (methods). The second contains data of ChEC-seq for the same variants with external spike-in for calibration, from which we extracted binding signals at spike-in promoters (methods). Shown on top is the scatter-plot (dots) and the linear fit (line) between those log2 transformed measures; the shaded area represents the Confidence Interval (CI) of the regression line. Note the strong negative correlation between them (p=-0.91). Shown on the bottom are the steps taken to calculate the absolute binding strength for mutants for which the spike-in experiment was not performed using the above-presented regression line (methods).

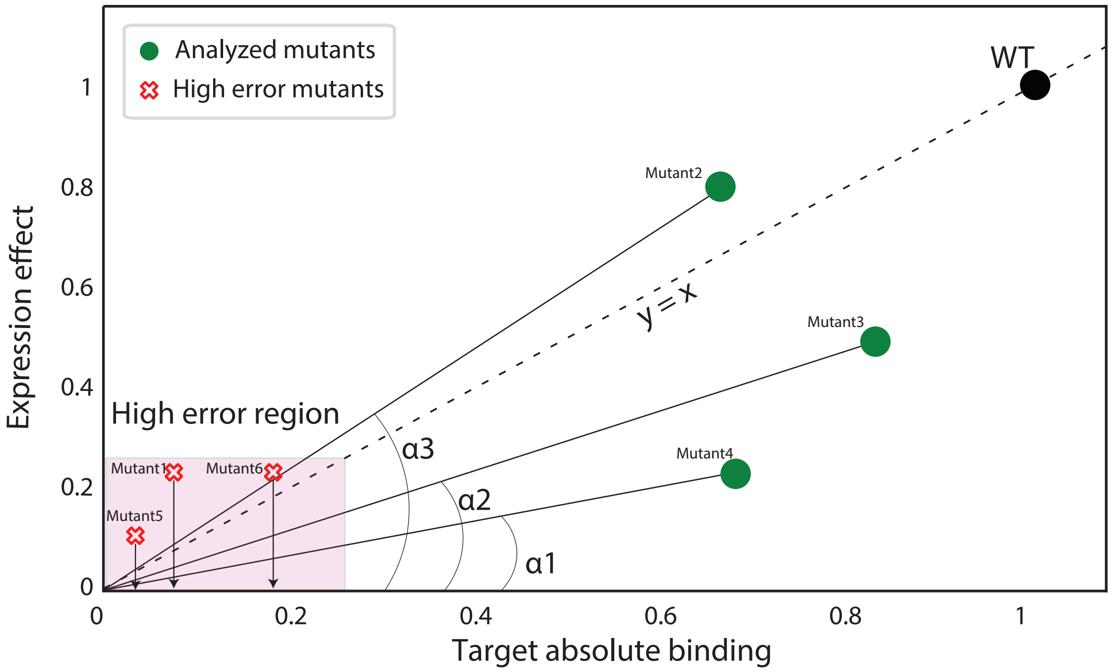
Analyzed mutants
High error mutants
WT
Mutant2
Mutant3
Mutant4
Mutant1
Mutant6
Mutant5
y = x
High error region
α3
α2
α1
Expression effect
0
0.2
0.4
0.6
0.8
1
Target absolute binding
0
0.2
0.4
0.6
0.8
1

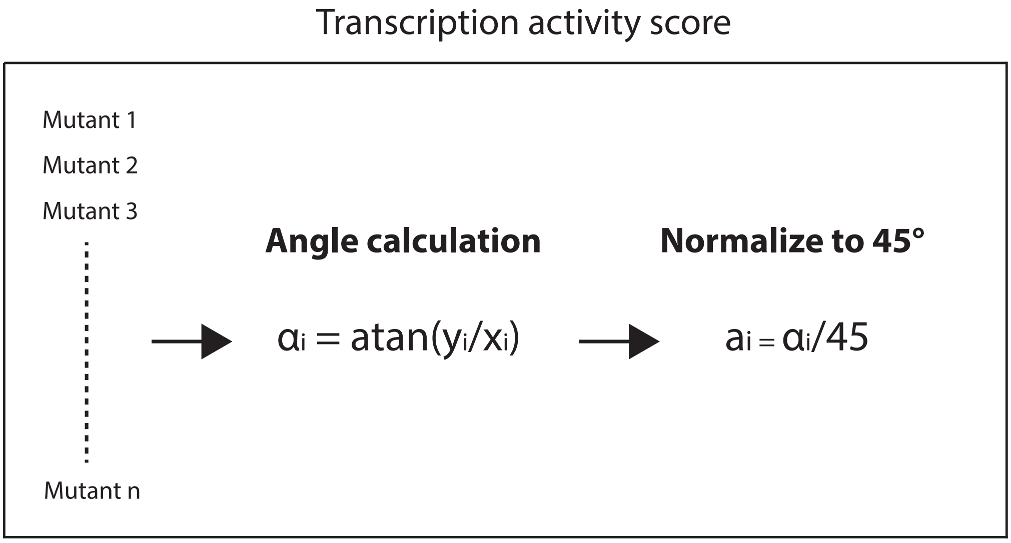
Transcription activity score
Mutant 1
Mutant 2
Mutant 3
Mutant n
Angle calculation
αi = atan(yi/xi)
Normalize to 45°
ai = αi/45

Figure S3. *Transcription activity score*

To calculate the transcription activity score, each mutant was positioned on the Target absolute binding (x-axis) and Expression effect (y-axis) graph (top, for calculation of the parameters above, see methods). Then, the angle between the line connecting the dot and the origin of the graph and the x-axis was calculated and normalized to 45 (with 45 being the angle of the WT, bottom, methods). Note that the area of low binding/expression <0.26 A.U (top, marked with red) is prone to yield high errors in the calculation of the transcription activity score; therefore, the scores of strains found within this area were set to zero.

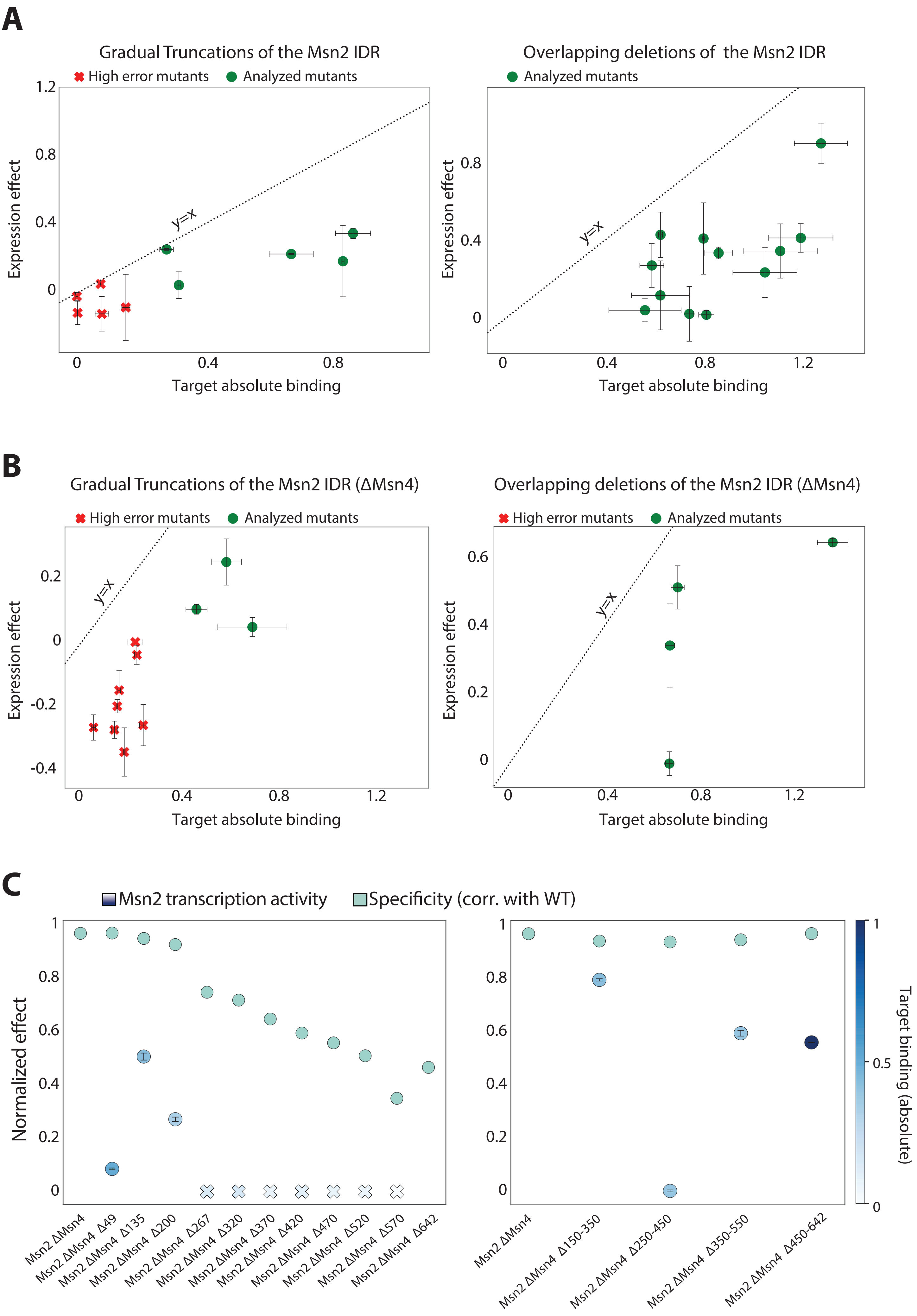
A
Gradual Truncations of the Msn2 IDR
High error mutants
Analyzed mutants
y=x
Expression effect
Target absolute binding
1.2
0.8
0.4
0
0
0.4
0.8
Overlapping deletions of the Msn2 IDR
Analyzed mutants
y=x
Expression effect
Target absolute binding
0.8
0.4
0
0
0.4
0.8
1.2
B
Gradual Truncations of the Msn2 IDR (ΔMsn4)
High error mutants
Analyzed mutants
y=x
Expression effect
Target absolute binding
0.2
0
-0.2
-0.4
0
0.4
0.8
1.2
Overlapping deletions of the Msn2 IDR (ΔMsn4)
High error mutants
Analyzed mutants
y=x
Expression effect
Target absolute binding
0.6
0.4
0.2
0
0
0.4
0.8
1.2
C
Msn2 transcription activity
Specificity (corr. with WT)
Normalized effect
1
0.8
0.6
0.4
0.2
0
Msn2 ΔMsn4
Msn2 ΔMsn4 Δ49
Msn2 ΔMsn4 Δ135
Msn2 ΔMsn4 Δ200
Msn2 ΔMsn4 Δ267
Msn2 ΔMsn4 Δ320
Msn2 ΔMsn4 Δ370
Msn2 ΔMsn4 Δ420
Msn2 ΔMsn4 Δ470
Msn2 ΔMsn4 Δ520
Msn2 ΔMsn4 Δ570
Msn2 ΔMsn4 Δ642
Msn2 ΔMsn4
Msn2 ΔMsn4 Δ150-350
Msn2 ΔMsn4 Δ250-450
Msn2 ΔMsn4 Δ350-550
Msn2 ΔMsn4 Δ450-642
Target binding (absolute)
1
0.5
0

Figure S4. *Msn2 transcription capacity depends on multiple components embedded within its long IDR*

Shown in (A) is the target expression effect as a function of the target absolute binding of gradual truncations (left) and internal deletions (right) of the Msn2 IDR. The error bars represent the Standard Error of the Mean between repeats (SEM, methods). Mutants for which the transcription activity scores were calculated are marked with green dots, and those found within high error regions with red crosses (methods, Fig. S3). (B) Same presentation as in (A) but for the strains deleted of the Msn2 paralog - Msn4. Shown in (C) is the transcription activity colored by a target binding (blue, methods) and a specificity measure (green, methods) for strains deleted of Msn4; strains marked by an “X” are in the high-error area and their transcription activity is set to 0. Note the strong effect of the Msn4 deletion on target gene expression (see methods for normalization).

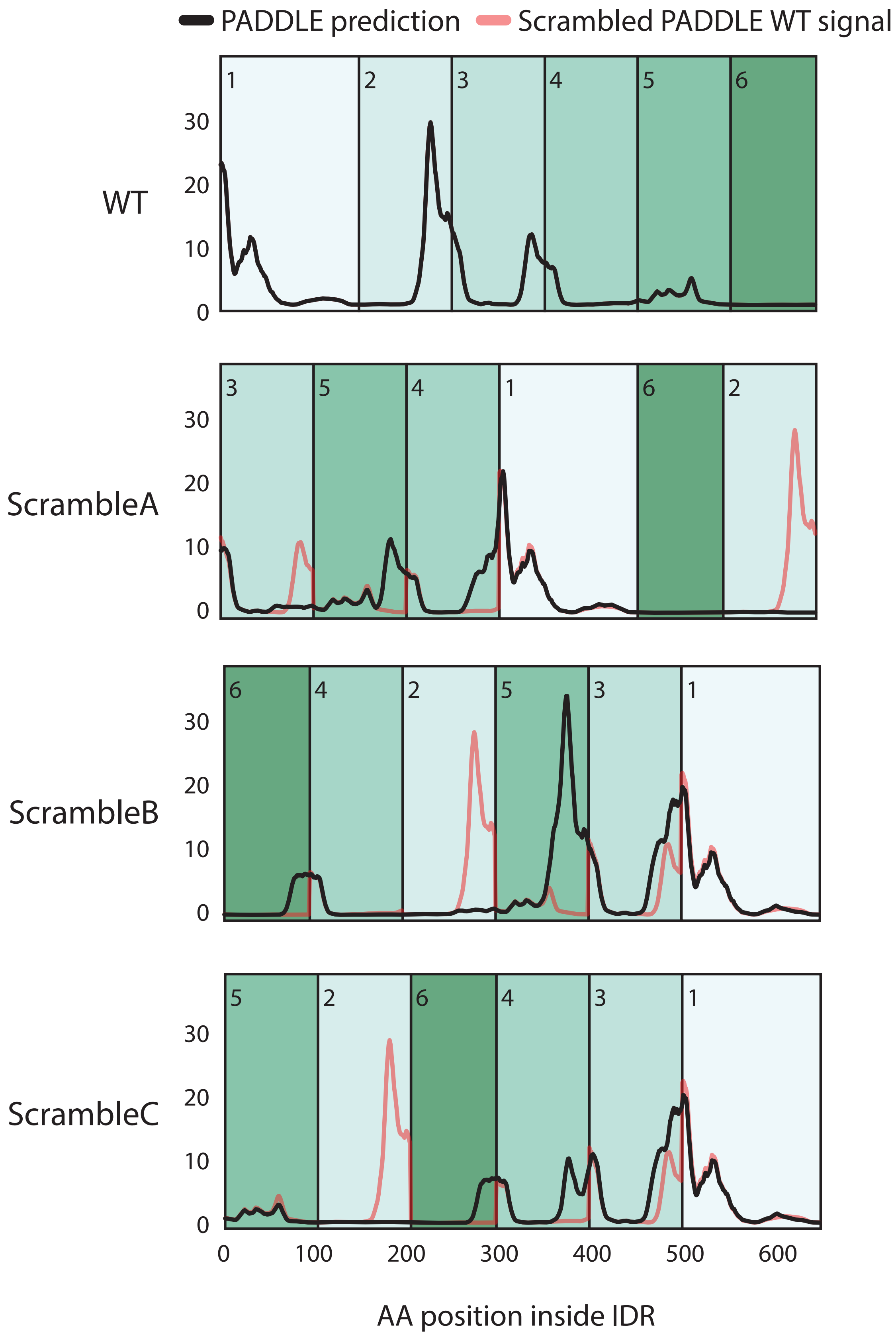

PADDLE prediction
Scrambled PADDLE WT signal
WT
ScrambleA
ScrambleB
ScrambleC
AA position inside IDR

Figure S5. *PADDLE activity prediction profiles of the scrambled IDR mutants*

The PADDLE prediction profiles of the WT Msn2 non-DBD and its Scrambled IDR mutations are shown. The different segments are denoted by the same color across all subplots. Black solid lines represent the PADDLE prediction of the respective variant. The red lines represent the predicted activity of each segment prior to scrambling.

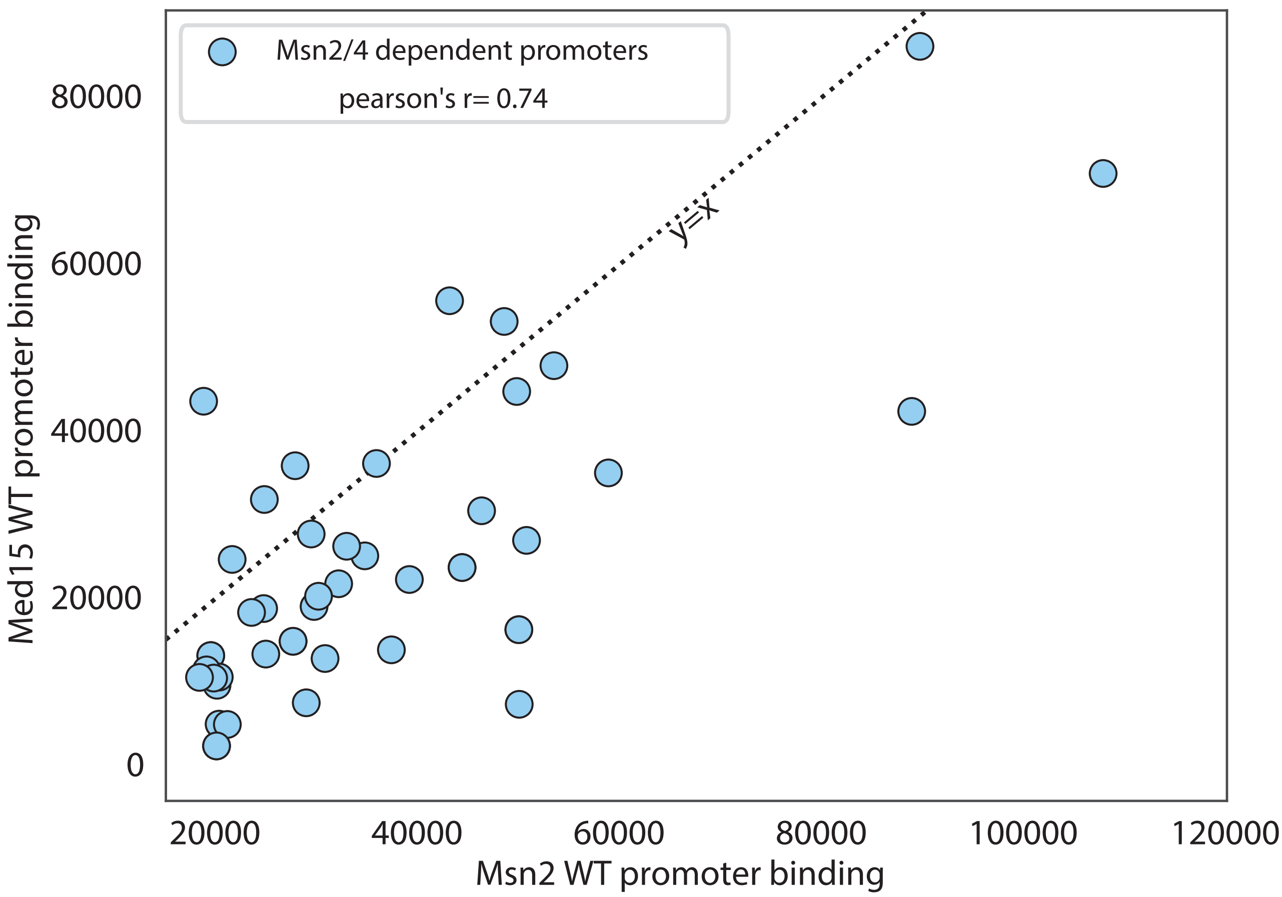

Med15 recruitment score

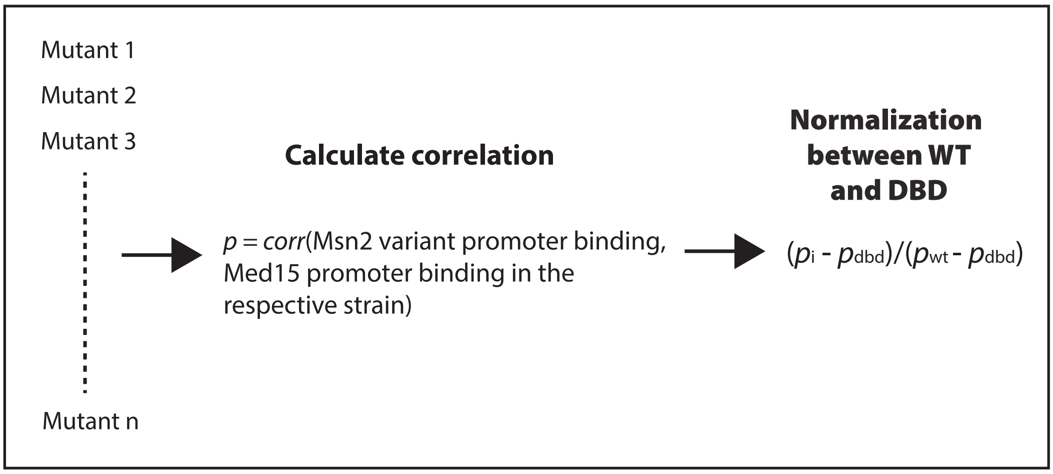

Figure S6. *Med15 recruitment score*

To define the Med15 recruitment score, the analysis focused on promoters on which Med15 depends on Msn2/4 (methods). The correlation of binding signals between each Msn2 variant and Med15 measured in the same strain on those defined promoters was calculated. An example measured in a wild-type *S. cerevisiae* strain is shown on top. Presented is the sum of signal on promoters measured for Msn2 (x-axis) and Med15 (y-axis), where each dot represents a promoter. The calculation steps performed for every strain to obtain the Med15 recruitment score (methods) are shown at the bottom.

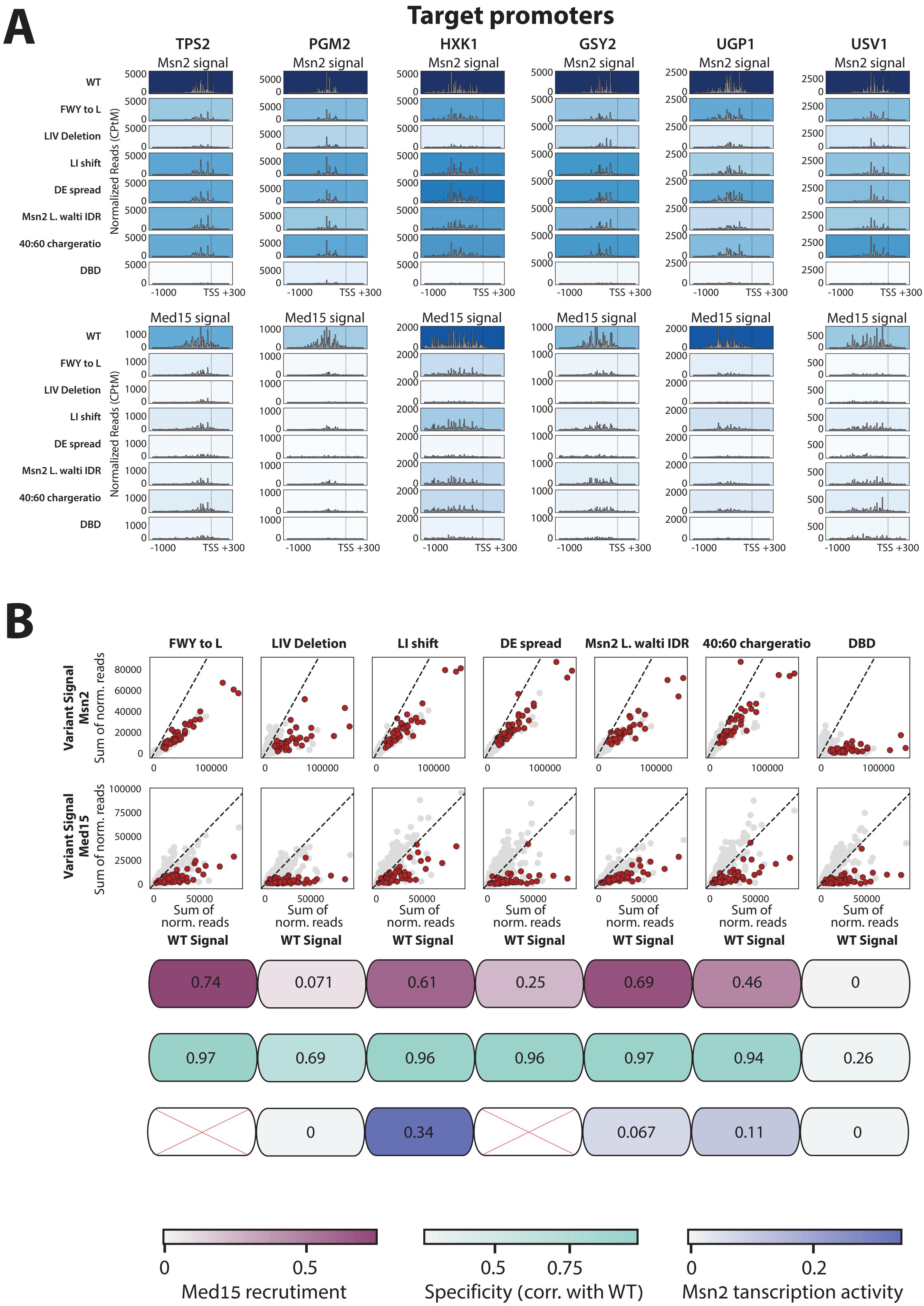
A
Target promoters
TPS2
PGM2
HXK1
GSY2
UGP1
USV1
Msn2 signal
Med15 signal
WT
FWY to L
LIV Deletion
LI shift
DE spread
Msn2 L. walti IDR
40:60 chargeratio
DBD
Normalized Reads (CPtM)
-1000
TSS +300
B
FWY to L
LIV Deletion
LI shift
DE spread
Msn2 L. walti IDR
40:60 chargeratio
DBD
Variant Signal
Msn2
Sum of norm. reads
Variant Signal
Med15
Sum of norm. reads
Sum of
norm. reads
WT Signal
0.74
0.071
0.61
0.25
0.69
0.46
0
0.97
0.69
0.96
0.96
0.97
0.94
0.26
0
0.34
0.067
0.11
0
Med15 recrutiment
Specificity (corr. with WT)
Msn2 tanscription activity

Figure S7. *ChEC-seq profiles of Msn2 and Med15*

Shown in (A, gray) are the CPtM (counts per ten million) normalized reads across 6 target promoters of Msn2. Shown on top is the binding signal of Msn2 and its indicated mutations. Shown on the bottom is the binding signal of Med15 in the strains bearing the respective Msn2 mutant. The transcription start site (TSS) is denoted by the dashed line. The sum of reads across the plotted region is color-coded on the background, with a higher signal having a deeper color for each promoter. Shown in (B, top) is a comparison of the sum of signal over each promoter across all promoters between the wild-type Msn2 and its indicated IDR mutations, and on (B, bottom) the binding signal of Med15 in wild-type cells compared to its binding in cells bearing the respective Msn2 mutation. Each dot represents a promoter. Marked with red are Msn2-dependent Med15 promoters (methods, Fig. S4). Med15 recruitment (burgundy), binding specificity compared to wild type (green), and transcription activity (blue) are shown as color-coded stripes on the bottom for each mutant. Denoted by red crosses are mutations for which RNA-seq profiling experiments are missing.

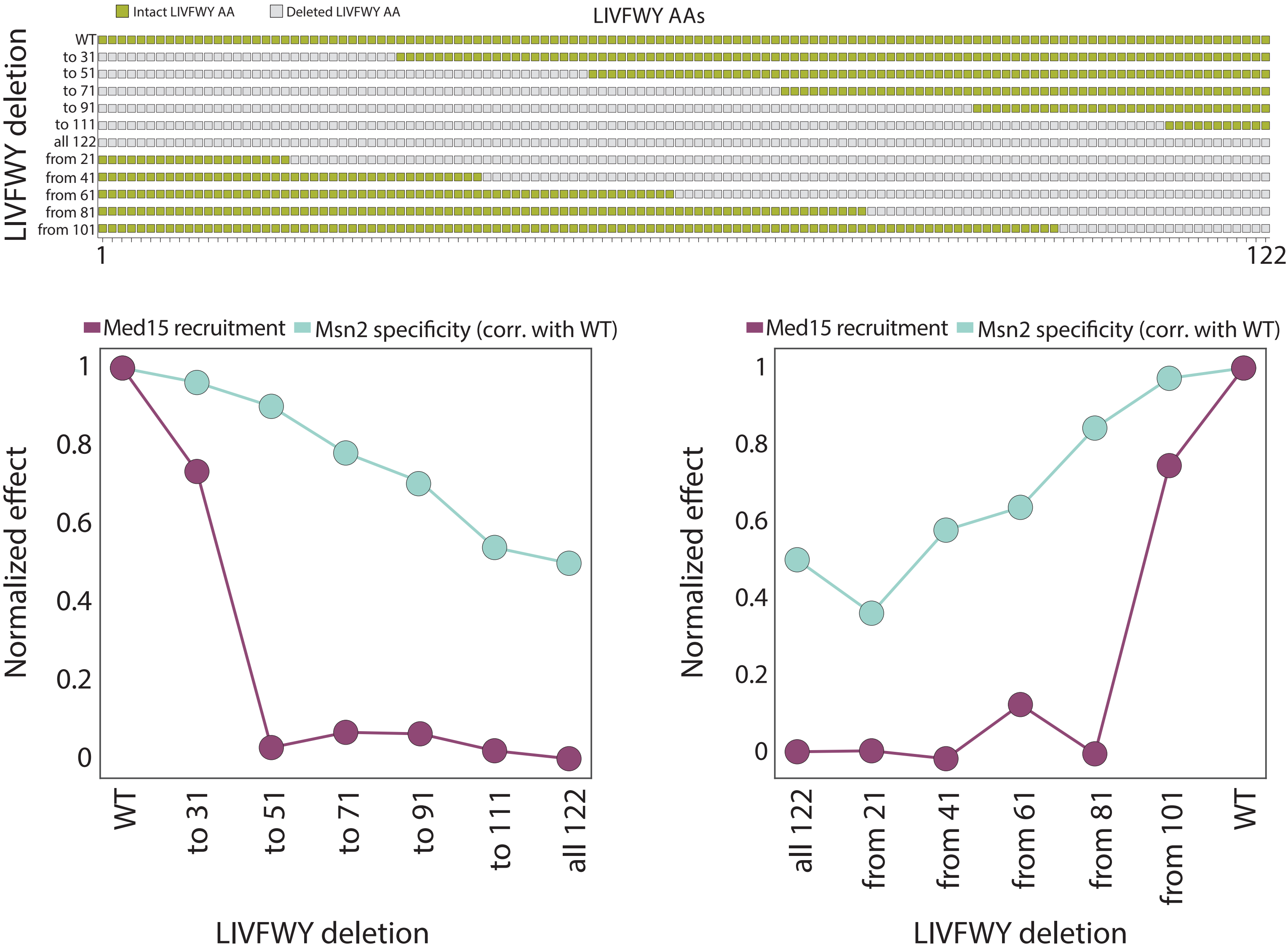

Intact LIVFWY AA
Deleted LIVFWY AA
LIVFWY AAs
LIVFWY deletion
WT
to 31
to 51
to 71
to 91
to 111
all 122
from 21
from 41
from 61
from 81
from 101
1
122
Med15 recruitment
Msn2 specificity (corr. with WT)
Normalized effect
1
0.8
0.6
0.4
0.2
0
WT
to 31
to 51
to 71
to 91
to 111
all 122
LIVFWY deletion
Med15 recruitment
Msn2 specificity (corr. with WT)
Normalized effect
1
0.8
0.6
0.4
0.2
0
all 122
from 21
from 41
from 61
from 81
from 101
WT
LIVFWY deletion

Figure S8. *Binding determinants are highly redundant, while transcription determinants are mutually required.*

A scheme of the analyzed strains (top). Shown for each strain are the remaining LIVFWY AAs (green) and the deleted ones (gray). Only the LIVFWY AAs in the Msn2 sequence are presented. The similarity in binding specificity to the wild-type Msn2 (green) and Med15 recruitment (burgundy) are shown (bottom, methods). Note that the loss of binding targets is gradual, while deleting short segments of hydrophobic AAs abolishes the capacity to recruit Med15.

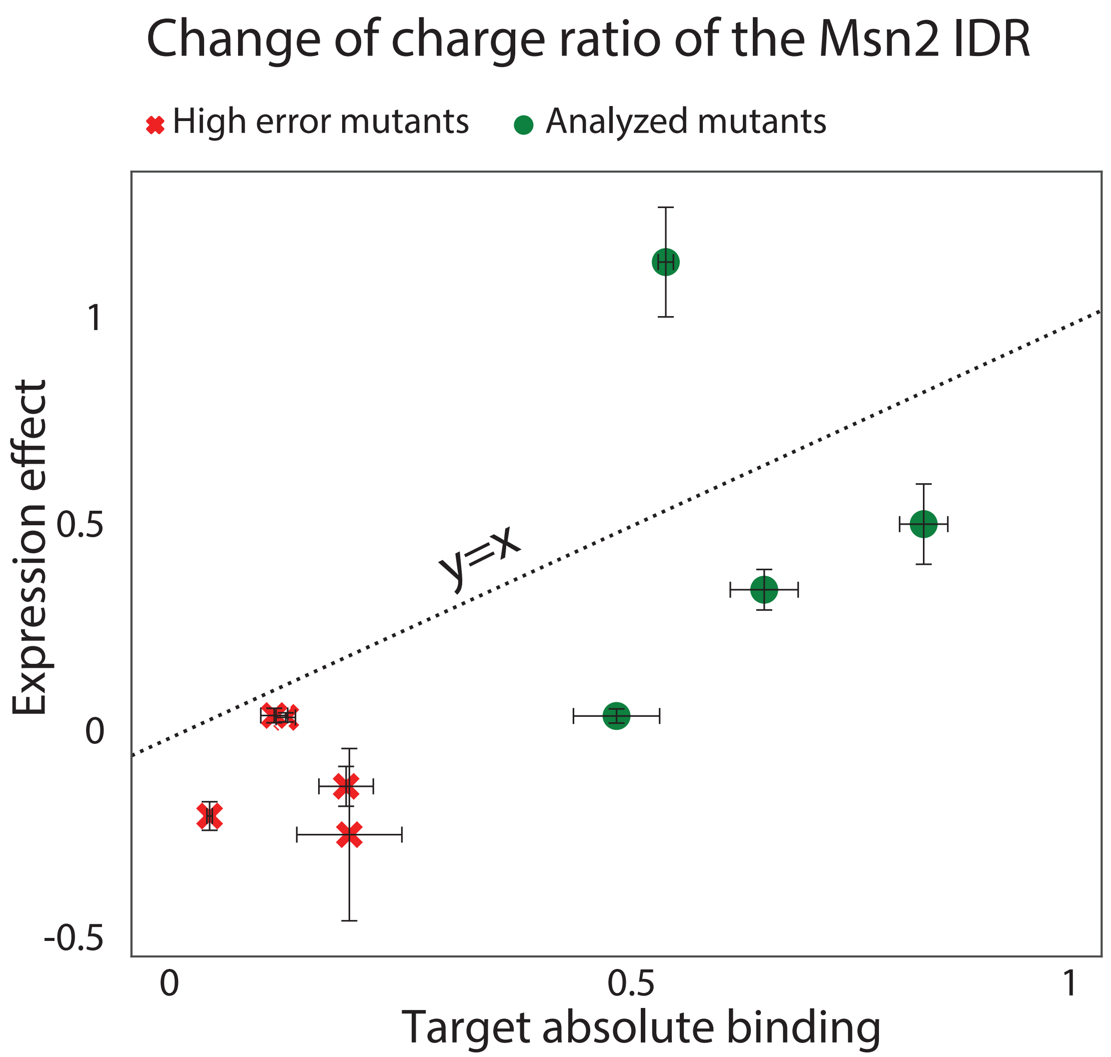
Change of charge ratio of the Msn2 IDR
High error mutants
Analyzed mutants
y=x
Expression effect
1
0.5
0
-0.5
0
0.5
1
Target absolute binding

Figure S9. *Recruitment of the Med15 coactivator by Msn2 requires multiple intrinsically disordered regions.*

Shown is the expression effect as a function of the absolute target binding for the series of Msn2 mutants in which the KR/DE ratio of the IDR was gradually changed. The error bars represent the Standard Error of the Mean between repeats (SEM, methods), analyzed mutants marked with green dots, and those found within the high error area with red crosses (methods, Fig. S3)

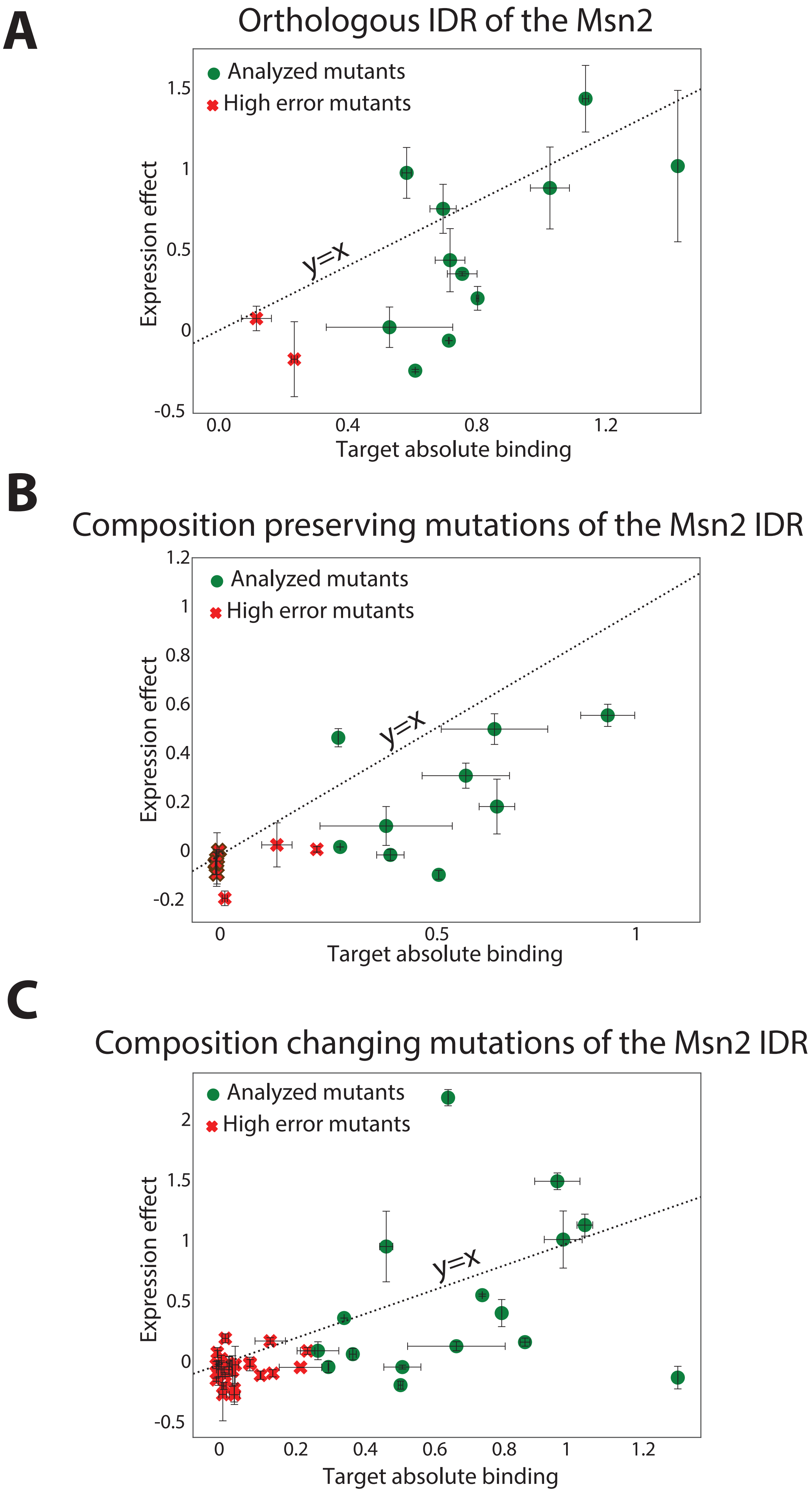
A
Orthologous IDR of the Msn2
Analyzed mutants
High error mutants
y=x
Expression effect
Target absolute binding
-0.5
0
0.5
1
1.5
0.0
0.4
0.8
1.2
B
Composition preserving mutations of the Msn2 IDR
Analyzed mutants
High error mutants
y=x
Expression effect
Target absolute binding
-0.2
0
0.2
0.4
0.6
0.8
1
1.2
0
0.5
1
C
Composition changing mutations of the Msn2 IDR
Analyzed mutants
High error mutants
y=x
Expression effect
Target absolute binding
-0.5
0
0.5
1
1.5
2
0
0.2
0.4
0.6
0.8
1
1.2

Figure S10. *Sensitivity of Msn2 transcription activity to IDR composition*

Same as in Fig. S4, for the datasets of orthologous Msn2 (A), composition preserving mutants (B, clusters of AA are marked with brown edges), and composition changing mutants of the Msn2 IDR (C).

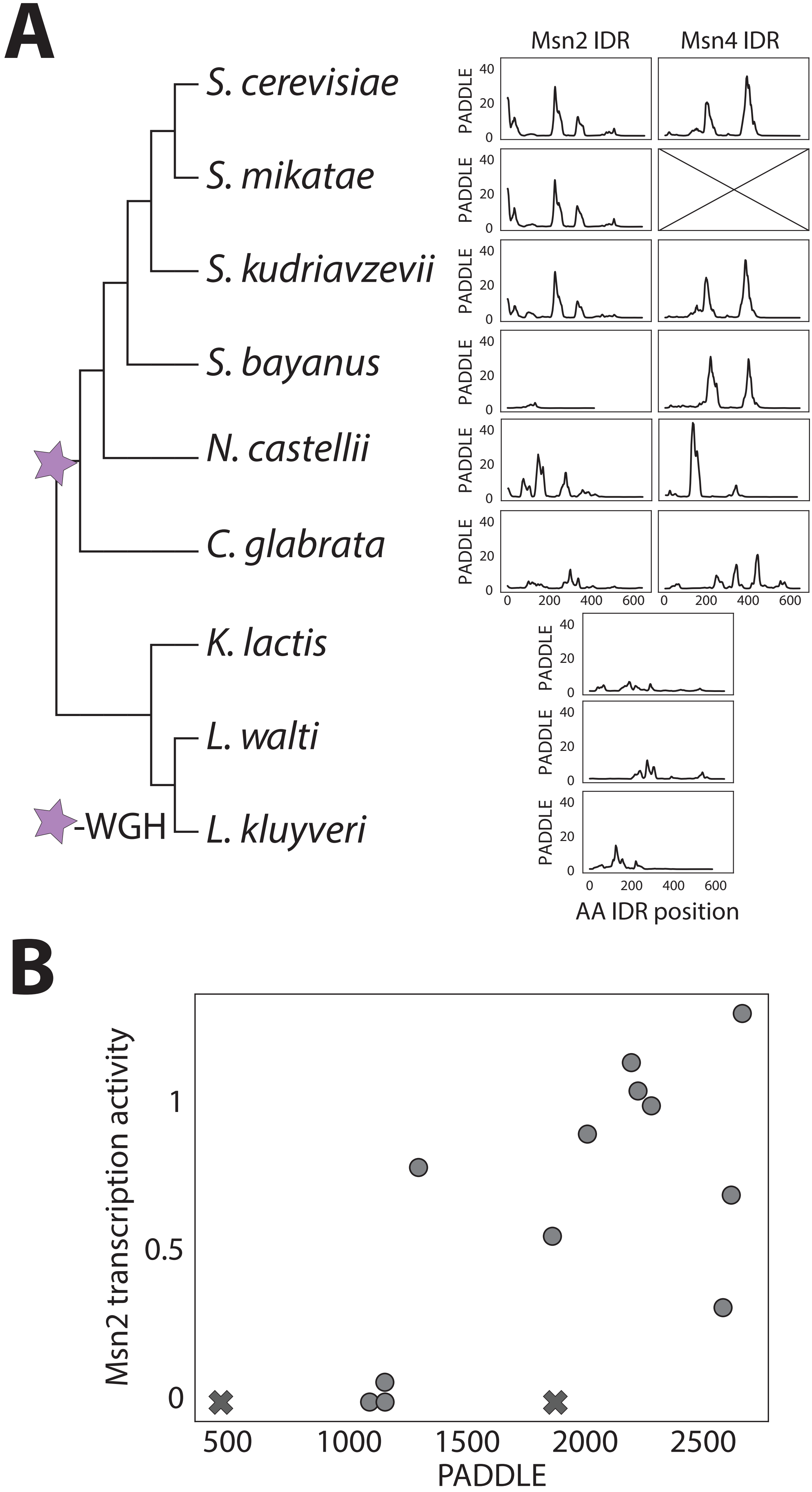

A
Msn2 IDR
Msn4 IDR
S. cerevisiae
S. mikatae
S. kudriavzevii
S. bayanus
N. castellii
C. glabrata
K. lactis
L. walti
L. kluyveri
-WGH
PADDLE
AA IDR position
B
Msn2 transcription activity
PADDLE

Figure S11. *PADDLE activation prediction profiles of Msn2/4 IDR orthologs*

Sown in (A) is a phylogenetic tree of the analyzed ortholog species as in (Fig. 5, left) and the PADDLE predicted transcription activity profiles of the analyzed IDRs from each species (right). Profiles marked by an “X” were not analyzed in this study. A comparison between the sum of the PADDLE predicted profiles from (A) and the transcription activity of the respective strains is shown in (B, methods). Strains found within the high-error area (low binding or expression) are denoted by “X” (Fig. S3, Fig. S10 A).

Table S1. *Detailed description of strains used in the study.*
